# Revealing invisible cell phenotypes with conditional generative modeling

**DOI:** 10.1038/s41467-023-42124-6

**Published:** 2023-10-11

**Authors:** Alexis Lamiable, Tiphaine Champetier, Francesco Leonardi, Ethan Cohen, Peter Sommer, David Hardy, Nicolas Argy, Achille Massougbodji, Elaine Del Nery, Gilles Cottrell, Yong-Jun Kwon, Auguste Genovesio

**Affiliations:** 1 Computational Bioimaging and Bioinformatics, Institut de Biologie de l’Ecole Normale Supérieure, PSL University, 46, rue d’Ulm, 75005 Paris, France; 2 Ksilink, 16 rue d’Ankara, 67000 Strasbourg, France; 3 Université Paris-Cité, MERIT, IRD, F-75006 Paris, France; 4 Histopathology Platform, Institut Pasteur, F-75015 Paris, France; 5 Laboratoire de parasitologie-mycologie, Hôpital Bichat-Claude bernard, APHP, Paris, France; 6 Institut de Recherche Clinique du Bénin, Abomey-Calavi, Benin; 7 Biophenics, Institut Curie, PSL Research University, Department of Translational Research, Cell and Tissue Imaging Facility (PICT-IBiSA), 26 rue d’Ulm, 75005 Paris, France; 8 Present Address: Personalized Therapy Discovery, Department of Oncology, Luxembourg Institute of Health, Dudelange, Luxembourg

**Keywords:** Image processing, Cellular imaging, Biomarkers, Organelles

## Abstract

Biological sciences, drug discovery and medicine rely heavily on cell phenotype perturbation and microscope observation. However, most cellular phenotypic changes are subtle and thus hidden from us by natural cell variability: two cells in the same condition already look different. In this study, we show that conditional generative models can be used to transform an image of cells from any one condition to another, thus canceling cell variability. We visually and quantitatively validate that the principle of synthetic cell perturbation works on discernible cases. We then illustrate its effectiveness in displaying otherwise invisible cell phenotypes triggered by blood cells under parasite infection, or by the presence of a disease-causing pathological mutation in differentiated neurons derived from iPSCs, or by low concentration drug treatments. The proposed approach, easy to use and robust, opens the door to more accessible discovery of biological and disease biomarkers.

## Abstract (web summary)

Biological research relies on observing cell phenotypes, often obscured by natural variability. Here, the authors use generative modelling to unveil hidden changes triggered by infections, mutations, or drugs, allowing for accessible discovery of biomarkers.

## Introduction

Assessing quantitative differences in visual cell phenotypes from microscopy images finds many applications in basic biological research, drug discovery, and medicine^1–3^. This task has historically relied on hand-crafted image analysis algorithms when the phenotype of interest was known and visible, and the cells could be segmented^4,5^. When phenotypes are too subtle to be discernible, a large list of quantitative features can still be computed (such as intensities, shape, texture or cell-to-cell relationships) from segmented cells. However, because these features are highly correlated and computed over a large number of cells, it is frequent (yet not necessarily intuitive) that in the case of subtle phenotypic variation, a large fraction of them display a statistically significant difference between conditions, while at a very low effect size. These two criteria indicate that a subtle phenotypic difference may be present, but identifying one of these features as relevant or deciphering biological meaning is difficult (see Supplementary Fig. 1 with examples of feature distributions for illustration in Supplementary Fig. 2, Supplementary Fig. 3, Supplementary Fig. 4). Ultimately, when the phenotype is too subtle to be discernible and the cells cannot be individually segmented to compute quantitative handcrafted features - as for instance when dealing with cancer cell colonies, neurons, complex tissues made of fibroblasts, muscle cells or intra- and intercellular structures with unclear boundaries - a deep Convolutional Neural Network (CNN) model can often still be trained in a supervised fashion from raw image tiles, in order to discriminate conditions with good accuracy, bypassing the cell detection step. This is the case for all conditions from all datasets in this paper (Supplementary Table 1). In other words, differences and similarities between cell phenotypes not discernible to the human eye can still, in many cases, be assessed by deep learning (DL), demonstrating that microscopy images contain much more information than what can be seen^6,7^. It is a great opportunity for biology and medicine to discover and investigate sounder, more subtle, and broader classes of phenotypes and biomarkers. However, as phenotype difference cannot be seen, DL approaches offer few options and no guarantee that discrimination is not based on a bias, an experimental artifact or a biological phenotype irrelevant to the considered assay. Conclusively, phenotype differences may be too subtle to be visually accessible. In this case, standard image analysis tools that cannot reliably detect objects such as cells are of little use. Furthermore, when these tools are able to measure quantitative differences that are visually indiscernible, the result often materializes into very low effect sizes observed on many features, which makes interpretation difficult. Ultimately, the measure of interest may not be part of the primary measurement offered by these tools. Ideally, one would first need to know what to measure in order to engineer a dedicated optimal image analysis algorithm, to properly perform an explainable and relevant measurement.

We propose here a straightforward method to address this issue, based on established work providing a general and accessible description of the differences between any two or more microscopy image sets. This allows us to infer invisible differences between conditions, and subsequently to draw hypotheses that could further be confirmed as relevant or rejected as uninteresting bias using real image data.

Our approach is based on the hypothesis that if two cellular visual phenotypes can be distinguished by a deep network, but not by the human eye, this is primarily due to the fact that cell-to-cell variability within an image largely overlaps cell-to-cell variability between phenotypes; hence the former hides the latter. In short, natural variability prevents us from seeing the subtle phenotype difference between two images of cells. We extrapolated that the subtle differences between two close phenotypes could be made visible and inferred if natural variability was canceled out. To this end, instead of comparing two real images of cells under conditions A and B that display necessarily different cells, we used a generative model to translate an image of cells under condition A to the image of the same cells under condition B. The signal modification thus produced could be defined as the difference between phenotypes induced by A and B. To implement this idea, we used conditional Generative Adversarial Networks (GANs).

GANs produced a drastic improvement in the image synthesis field by taking advantage of an adversarial training scheme occuring between a generative network and a discriminative network^8^. This principle has since been widely used and improved in many ways, in order to generate various kinds of data. Numerous compelling works in image generation and translation have been proposed but never to our knowledge with the aim of explaining invisible changes between conditions^9–13^. On the contrary, because the aim is different, domains chosen for such work were usually visually different in order to validate the approaches.

In this study, we demonstrate that conditional image synthesis can artificially replicate the effects of various perturbations on a cell image. Furthermore, we provide quantitative evidence, based on three assays and approximately 50 conditions, to support the reliability of synthetic features in reproducing real ones. We also show that quantitative distinctions between real conditions can be accurately inferred from synthesized ones. Finally, we illustrate that this approach opens possibilities for deciphering subtle variations of phenotypes triggered by a parasite infection on blood cells, a mutation in human neurons, or a low concentration of compound treatment, all invisible to the human eye.

## Results

### Conditional GAN can synthesize cell phenotype perturbations

**Fig. 1:**
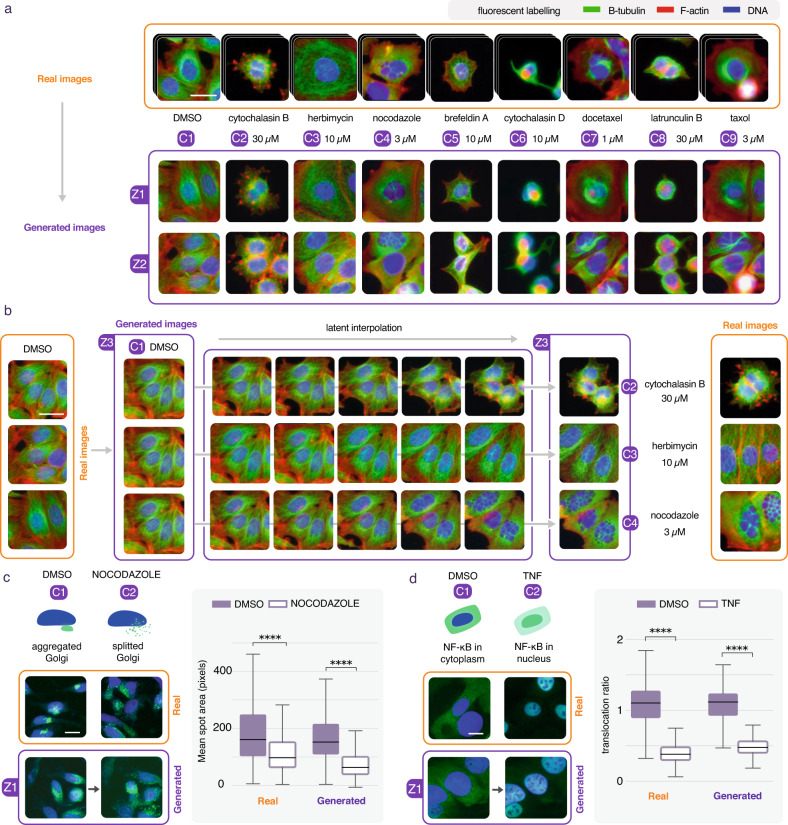
Conditional synthesis of cell phenotype perturbations. **a** A conditional GAN is trained on real images (orange) of DMSO and high concentration drug treatments (C1, C2, C3, etc.), scale bar is 20 μm. Synthetic images (violet) of these treatments can be generated from the same random seed, here z1 or z2. The high compound concentrations of these examples make the phenotypic changes obvious and visible. Surrounding cells in the negative control (DMSO) are removed from the images of compound treatments because of their toxicity. **b** A latent traversal can be computed for a single seed (z3) from the untreated state (C1 = DMSO) to 3 different treatment effects (C2,C3,C4) displaying each a different gradual change of the same cells. **c** A standard assay such as the nocodazole induced golgi scattering (green) can be reproduced with synthetic images, scale bar is 20 μm. An image analysis measurement (mean spot area) performed on real and synthetic images of both conditions led to the same quantitative conclusion (*n* = 1000 for each sampled condition, two sided *t*-test. Real: *p* = 5.1e-19, *T*(1998) = 9.0, confidence interval(99.99%) = [43.05, 108.84], Cohen’s *d* = 0.403. Generated: *p* = 5.35e-113, *T*(1998) = 24.12, Confidence interval(99.99%) = [78.0, 108.02] Cohen’s *d* = 1.079, ****p-value < 0.0001). **d** Another standard assay displaying TNF-induced NFkB translocation (green) can also be reproduced, scale bar is 20 μm (*n* = 1000 for each sampled condition, two sided *t*-test. Real: *p* = 0, *T*(1998) = 178.2, Confidence interval(99.99%) = [0.66, 0.69], Cohen’s *d* = 2.87. Generated: *p* = 0, *T*(1998) = 60.73, Confidence interval(99.99%) = [0.53, 0.60], Cohen’s *d* = 2.75, ****p-value < 0.0001), *p* values were not adjusted. Boxes represent the q1-q3 interval (25–75% of the distribution). The central bar is the median. The lower whisker is the first datum greater than q1 − 1.5 × IQR and the upper whisker is the last datum lower than q3 + 1.5 × IQR. IQR is interquartile range. Source data are provided as a Source Data file.

We trained a conditional GAN (see online methods) to reproduce the transformations - or translations - that cells undergo when subject to various high-dosage compound treatments. In this way, using a single model and a single training, we could artificially produce images of the effect of various compound treatments, when applied to a single image of untreated cells. The real images of Fig. 1a, b were taken from the Broad Bioimage Benchmark. It consists of an assay of Human MCF-7 breast cancer cells treated for 24 h with small molecules at various concentrations. After fixation, cells were labeled for F-actin, B-tubulin, and DNA, and imaged by fluorescent microscopy, as described in^14^. In this first training, images of treated cells were chosen so as to display an obvious phenotype at high concentration (Supplementary Fig. 5). Results demonstrated the capability of conditional image generation to reproduce cell phenotypes induced by compound treatment (Fig. 1a). Furthermore, synthesis could be performed for all treatments from the same cell image, and latent traversals enabled us to see gradual changes that need to be performed in order to transform an untreated (DMSO) cell to any compound treated cell (Fig. 1b). We engineered a web interface to ease visualization and manipulation of such data on many examples for all datasets considered in this work (data can be manipulated here https://www.phenexplain.bio.ens.psl.eu/hd.html and here https://www.phenexplain.bio.ens.psl.eu/lda_translation.html). Interestingly, beyond the phenotypes themselves, the synthetically treated cell images displayed a lower cell count, which is consistent with the fact that these treatments are toxic at high concentration (Fig. 1a, b and Supplementary Fig. 6). Notably, as intermediate generated images are also validated by the conditional GAN discriminator as possible images from the dataset, cells on the border do not gradually disappear, but tend to gently shift out of the field of view. This last example demonstrates that this type of morphing doesn’t match any sort of real dynamic. However, it does certainly help to visualize the differences between phenotypes, especially when these are subtle.

### Synthetic cell features match those of real treatment

To quantitatively evaluate the ability of conditional image generation to properly reproduce real phenotypes, we computed the Frechet Inception Distance (FID), a commonly used metric to assess the quality of synthetic images produced by a generative model^15^. In short, the lower the FID value the better, since it is defined as a distance between a sample distribution of real image representations and a sample distribution of synthetic image representations. The FID values obtained for all assays presented in this paper were particularly low compared to those obtained with natural images, indicating a very high image quality, closely reproducing the real distributions of cell image data (Supplementary Table 2 and Supplementary Fig. 7). We furthermore computed ~213 CellProfiler quantitative features (monitoring for intensity, shape, and texture) on real and synthetic images of 50 conditions (7 compounds each at 7 concentrations and DMSO). Results demonstrate that synthetic and real cell features are highly correlated across compound concentrations (Supplementary Fig. 8). We applied our approach to two other assays to verify if differences measured between conditions on real images could still be assessed on synthetic ones. The first assay monitors the morphology of the golgi apparatus state. By treating cells with nocodazole, microtubules are depolymerized and the golgi originally located at approximately the center of the cell is split into mini stacks (Supplementary Fig. 9). A simple average spot size difference measured on 1000 real treated and 1000 untreated images could be reproduced when computed only from synthetic images (Fig. 1c). The second assay monitored the subcellular location of NF-kB (nuclear factor kappa B) protein. When treated with TNFα (pro-inflammatory cytokine tumor necrosis factor alpha) the transcription factor translocates to the nucleus, and the corresponding fluorescence signal moves from the cytoplasm to the nuclear area, with cells displaying bright green nuclei (Supplementary Fig. 10). Similarly, for this assay, the difference in nucleus versus cytoplasmic fluorescence ratio per cell measured on 1000 real treated and 1000 untreated images could be very closely reproduced when computed from synthetic images (Fig. 1d). Confirming that quantitative features difference on real images could be reproduced on synthetic images for these assays thus suggested that imperceptible yet reliable phenotype variations could also be reproduced.

### Deciphering blood cell changes triggered by a parasite infection

**Fig. 2:**
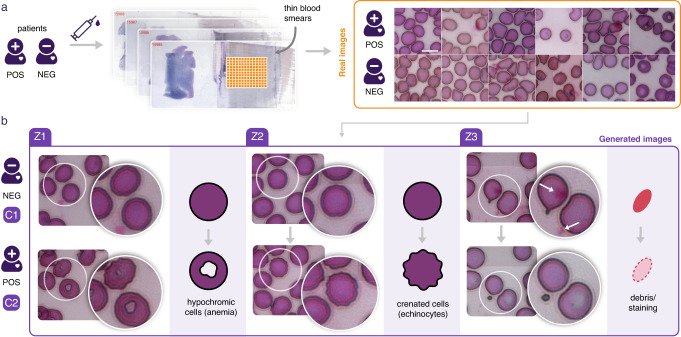
Unraveling red blood cell morphological changes related to an infectious environment. **a** Images of blood cells were extracted from thin blood smears sampled from a population of people exposed to malaria. In all, 200 slides were selected as negative for Malaria by microscopists, meaning that no parasites could be found on any of these slides, with nevertheless half of them found to be positive by qPCR. Note that on both qPCR positive and qPCR negative slides, images extracted displayed variable cell densities with variable background, did not contain any parasites, and did not show any identifiable systematic visible differences, scale bar is 10 μm. **b** In order to identify discriminative features between qPCR+ and qPCR- slides, we used 60,000 such images from these 200 slides to train a conditional GAN. This panel shows three representative generated images of the results found. Z1 displays a visual difference that can be interpreted as an increase of anemia: the content of some blood cells lose hemoglobin (displayed as a hole or a white halo in the cell). This phenotype could barely be identified from real images data because both qPCR+ and qPCR- slides contain anemia cells. Additionally, Z2 displays some deformations of the cell membrane producing crenated cells. Finally, Z3 shows that the negative sample contained more debris due to staining as a translation to qPCR+ tends to remove these artifacts. Indeed, the system cannot discriminate between relevant differences of phenotypes from biologically irrelevant differences related to possible technical or experimental biases.

The gold standard diagnostic method performed on patients with suspected malaria in endemic areas is based on microscopic observation of blood film to detect intraerythrocytic *plasmodium* spp. This method presents the compelling advantages of being inexpensive, rapid, not requiring any advanced device and usable directly on the field, close to the patients. However, this approach is also less sensitive than molecular ones such as qPCR. While qPCR is more expensive, necessitates lab work and an expensive device, and is not available on the field, it can reveal “submicroscopic active infection”. This is defined as a qPCR positive result while no visible parasites were found by microscopic examination and it can represent up to half the positive cases in some areas^16,17^. We then aimed to evaluate the hypothesis that such infection could still be detected from microscopy images despite the absence of visible parasites. This would pave the way to a possible fast and low-cost microscopy-based sensitive diagnostic test available on the field. We then collected thin blood smears and first selected 100 slides that were diagnosed positive for plasmodium infection by qPCR but did not display any visible parasites, thus classified as negative by a microscopist, then selected 100 slides that were diagnosed negative by qPCR, therefore non-infected patients (Fig. 2a). 300 images were extracted from each of these 200 slides (Supplementary Fig. 11). Intriguingly, when performing computation of 172 Cell profiler features on single cells detected from these images, about half of these highly correlated feature distributions were significantly different between conditions but they all had a very low effect size. Therefore it was not helpful to select one explanatory feature nor helpful to infer morphological variations that could distinguish the qPCR+ from the qPCR- cases (see Supplementary Fig. 1C). Similarly, training a simple convolutional neural network showed that classification of these slides could be achieved with 74% accuracy (Supplementary Table 1), indicating that it contained invisible discriminative features. We then trained a conditional GAN on these close phenotypes. Results show that transforming an image of qPCR- to qPCR+ necessitates generation of more anemia and more crenated cells (see examples here https://www.phenexplain.bio.ens.psl.eu/malaria.html). In effect, the translation from a blood cell of a healthy patient to the blood cell of an infected patient seems to erase part of the hemoglobin content which, while not specific to the malaria infection, tends to denote an infectious context^18,19^. We also found that some cells were transformed into crenated cells which were previously reported to be related to certain liver diseases^20^. Finally, the system also detected that there might have been a relative excess of color staining in some of the negative slides because the synthetic translations to the positive case showed a lower amount of debris due to the staining step (Fig. 2b). With these observations in mind, we trained two dedicated CNNs on the real images to distinguish normal cells from crenated or hypochromic cells (independent from the conditions). Projecting all the cells from the two conditions on these two engineered discriminative axes (obtained from computing a Linear Discriminant Analysis - LDA - on the last layer of these CNNs), we could confirm from the real images that subpopulations of these specific cell phenotypes were indeed slightly shifted, suggesting that variation between conditions could be due to a slight change in these cell phenotype distributions (Supplementary Fig. 12).

### Uncovering morphological variations in patient-derived dopaminergic neurons

**Fig. 3:**
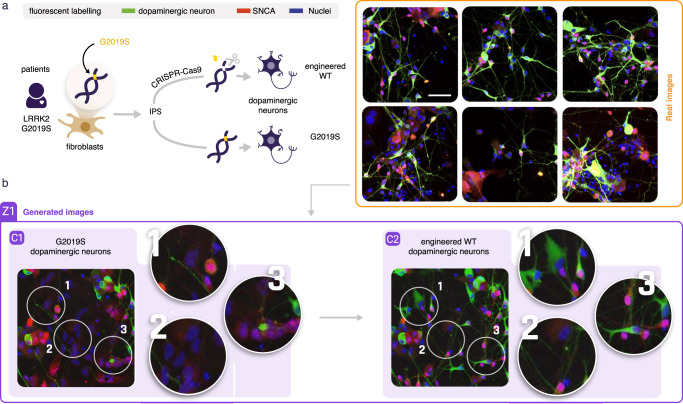
Unraveling invisible morphological variation in a patient-derived dopaminergic neuron assay. **a** IPSCs are derived from fibroblasts sampled from a LRRK2 G2019S mutated Parkinson’s patient. These IPScs are then reprogrammed to dopaminergic neurons with or without a CRISPR-cas9 correction of the G2019S mutation. The latter is an isogenic engineered wild type. Large sets of confocal images with one dye labeling for Nuclei, and antibody stains labeling for alpha-synuclein and TH cells, scale bar is 20 μm. Real images display no detectable visual systematic differences. **b** A conditional GAN trained in order to identify differences between these two close conditions displays 1 - an increase of dopaminergic neurons and dendritic complexity in the engineered WT condition, 2 - removal of some of the IPScs in the WT condition, 3- alpha-synuclein seems to shift from the IPSc cytoplasm to the nuclei before it eventually decreases in fully shaped differentiated WT neurons (more examples here https://www.phenexplain.bio.ens.psl.eu/lrrk2.html).

We subsequently wondered if this approach could be used to investigate subtle morphological variation induced by a mutation. We used iPSCs reprogrammed from fibroblasts of a Parkinson’s disease (PD) patient carrying the LRRK2 G2019S mutation along with an isogenic control, in which the mutation was genetically rescued to the wild-type sequence by gene editing and differentiated the iPSCs into dopaminergic neurons^21^. The LRRK2 G2019S mutation, localized in exon 41 of the LRRK2 (leucine-rich repeat kinase 2) gene, is causally linked to the development of Parkinson’s Disease^22^. Therefore, such a high content assay could be used to identify drugs that tend to rescue the wild-type phenotype (Fig. 3a). After immunostaining for nuclei, Tyrosine Hydroxylase (TH, a marker for dopaminergic neurons) and alpha-synuclein (SNCA, a protein that accumulates in Lewy bodies and Lewy neurites in Parkinson’s disease and other synucleinopathies), we acquired images of isogenic dopaminergic neurons with and without the G2019S mutation. However, these two conditions looked indistinguishable (Supplementary Fig. 13). As segmentation with common image analysis tools could not effectively detect neuronal cell bodies, features extracted from these segmentations again displayed very similar distributions between the two conditions, preventing interpretation (Supplementary Fig. 1B). A convolutional network could, however, discriminate them with 63% accuracy at the single image level and with 100% accuracy when aggregated at the well level (Supplementary Table 1). Training a conditional GAN on these two image sets highlighted differences between conditions from which interpretation was possible (Fig. 3b). The generated images of engineered WT cell cultures displayed an increase of dopaminergic neurons and neurite length, a decrease of non-differentiated cells and a displacement of alpha-synuclein from the cytoplasm to the nucleus before it decreases in the fully differentiated WT neurons as compared to the LRRK2 G2019S cultures (see examples here https://www.phenexplain.bio.ens.psl.eu/lrrk2.html). As for the previous examples, we could train dedicated CNNs on real images to discriminate non neuron from neuron cells, and discriminate neurons with short and long neurites. Projecting both conditions to these phenotypic axes confirmed a 10% difference in neuron to non neuron cell ratio and an increase in neurite length (Supplementary Fig. 14). From these observations, several hypotheses could be drawn. The slight difference in neuron to non neuron ratio could denote an effect of the mutation or an experimental bias that would lead to higher cell death or lower differentiation efficiency. While the suggested approach can make invisible differences visible and in this way enable us to suggest hypotheses, obviously it cannot decipher the cause of these differences which would require additional experiments. Interestingly, our approach confirmed that our assay could recapitulate previously made observations on the effect of the mutation, such as a decrease of neurite complexity and an increase of alpha -synuclein in fully differentiated neurons^23^.

### Low drug concentration effects can be made visible

**Fig. 4:**
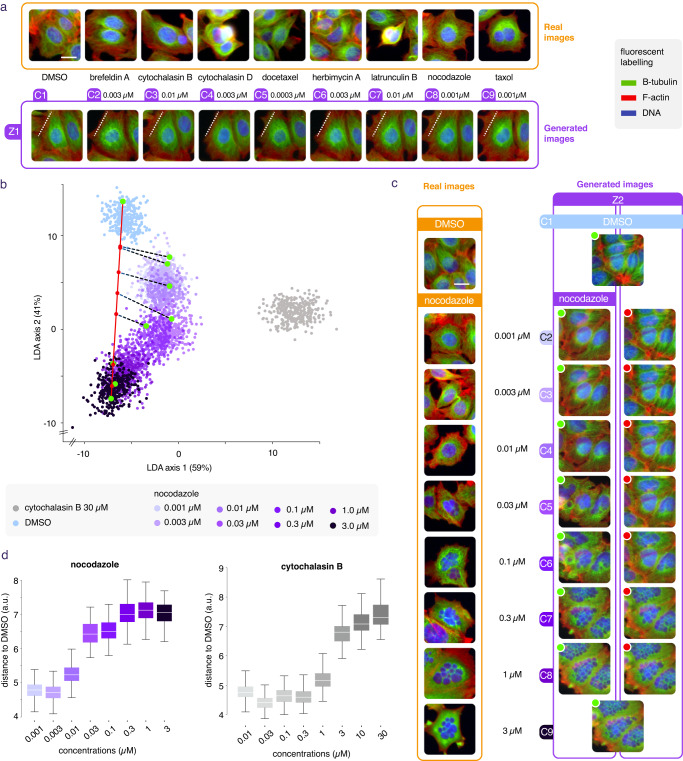
Morphological effect of perturbations by low dose compound treatments and dose response. **a** The same compounds used at high concentration in Fig. 1 were also plated at very low concentrations. The corresponding images cannot be visually distinguished from untreated cells (DMSO) and from one another (first row), scale bar is 20 μm. A conditional GAN was trained on real images of these low concentration compound treatments. By doing so we could generate artificial images of these perturbations on the same cells and compare them with DMSO and with each other (second row). We see that most treatments, even at low doses, had a slight toxic effect as they removed cells on the image borders compared to DMSO. Furthermore, some compounds tend to expand the cell cytoplasm while some others contract it. **b** An LDA plan is computed from generation of 3000 DMSO, 3000 Nocodazole and 3000 cytochalasin B at highest doses. Then, 300 samples of each available concentration of the Nocodazole treatment were drawn and projected onto this plan. **c** Left column, real images from a nocodazole dose response, middle column, z2 is a random seed used to generate perturbations of the same cells for each concentration (green dots on panel **b**) and, right column represents their orthogonal projections on the latent traversal between DMSO and the highest nocodazole dose in the W space: red dots on the red axis. **d** Computation of the distances in the W space of *n* = 1000 samples from each dose (C2, C3, etc.) to the DMSO (C1) for a given compound enables construction of a dose response curve describing the gradual intensity of the morphological changes. Boxes represent the q1-q3 interval (25–75% of the distribution). The central bar is the median. The lower whisker is the first datum greater than q1 − 1.5 × IQR and the upper whisker is the last datum lower than q3 + 1.5 × IQR. Source data are provided as a Source Data file.

We then used the same approach to display the invisible morphological changes that could be induced by low concentration drug treatment. To this end, we considered images with cells treated at very low concentration of the same drugs used for Fig. 1. Due to cell variability, the real images of these treatments displayed no visible differences, both compared to the untreated cells and between one another (Supplementary Fig. 15). Nonetheless, for most of these treatments, a simple convolutional network could be trained to distinguish both cases (Supplementary Table 1). We then trained a conditional GAN to translate images of untreated (DMSO) cells to images with cells treated with a low concentration of these drugs (Fig. 4a). Results showed that any of these treatments, even at the lowest available concentration, had a slight toxic effect, as a few cells on the border of the images were systematically removed. We could confirm on real images that the fraction of background was related to the cell toxicity by plotting one against the other in 50 conditions (Supplementary Fig. 6). Furthermore, some treatments, such as cytochalasin D, seemed to systematically contract cytoplasm, while others, such as taxol or nocodazole, extended it (additional examples can be seen here https://www.phenexplain.bio.ens.psl.eu/ld.html). Remarkably, we could confirm this visual clue on real images of low concentration drug treatments using the cytoplasm area value, a feature directly available this time from Cell profiler (Supplementary Fig. 16). This confirmation demonstrated that engineering a dedicated measurement or training a CNN (such as we did with the two previous examples) to confirm phenotypic changes on real images was not systematically needed if already available. In this case, the method could still be useful to designate (and visualize) a clear and interpretable change among hundreds of highly correlated low effect size features.

### Dose response can be approximated by latent traversal

The sequence of images generated from the latent interpolation between two conditions could not be considered as matching any dynamic reality because no dynamic data were provided to the system at training time. We did wonder whether it could approximate treatments at variable concentrations, or a so-called dose response. To evaluate this, we computed a Linear Discriminant Analysis (LDA) from 3000 data points generated for each of 3 extreme conditions: nocodazole and cytochalasin B at highest concentrations and DMSO. We then projected 300 data points from all concentrations of Nocodazole on this discriminative plan (Supplementary Fig. 17). The result shows that the consecutive concentrations do form a clear path on the discriminative plan from DMSO to the highest dose (Fig. 4b). We could then, for a given seed z, display side by side the generated sequence conditional to each dose on one hand, and their orthogonal projection (in the high dimensional space W) on the latent traversal on the other hand (Fig. 4c). The images thus retrieved for several compounds suggested that the latent linear interpolation between DMSO and a high dose was a reasonable approximation of the dose response (data can be manipulated here: https://www.phenexplain.bio.ens.psl.eu/doseresponse.html). We then measured the distance in the latent space between the untreated cells (DMSO) and each generated dose of nocodazole and cytochalasin B (Fig. 4d). It is compelling that the lowest doses could already be discriminated from DMSO, and the distance to DMSO increased with the concentration of compound, suggesting a method to quantify the relative amplitude of the effect of compound treatments on a considered assay, without performing any specific measurement.

## Discussion

Understanding the subtle effect of genetic, chemical, or disease-based perturbation on cell phenotypes is instrumental to cell biology. In this work, we suggest that cell variability is the main barrier to visually assessing the subtle effects produced by a perturbation on cells or simply the difference between two close conditions or mutants. We thus propose an approach based on conditional GAN to make the translation of cell images between two close phenotypic conditions possible, and have quantitatively assessed that the difference obtained corresponds to the actual modification. We demonstrated the advantage of such a method to decipher fine phenotypic alteration triggered by an infection, a mutation on patient-derived neurons, low concentration of drugs or even otherwise invisible biases. We further showed that the latent space thus created enabled quantitative comparison between close imperceptibly different perturbations, such as low doses of a compound treatment, and permitted the approximation of a dose response.

An alternative solution to consider would consist in first training a supervised classifier on two classes, then using tools such as class activation maps to explain what the classifier has learnt. Class activation maps essentially point to areas where visible discriminative features learnt by a classifier lay on an image^24^. However, when these features are invisible, as with subtle cell phenotypes, these methods become inefficient and cannot lead to interpretation, as shown on our own data (Supplementary Fig. 18). They could not lead us to use real images to even partially reach the clear observation we could make otherwise from synthetic ones. Some recent work has used generative models to explain what a classifier learnt in order to display already perceptible or known features (that were actually used to annotate the pictures), yet to our knowledge, these were never evaluated on invisible cell phenotypes in the context of various assays^12,13^. Furthermore, our approach does not focus on explaining a trained classifier and thus does not require one. On the contrary, these methods attempt to explain what a classifier can discriminate against, but do not directly explain the differences between datasets. While these goals are different, using a trained classifier toward our end goal would add a non neutral intermediate proxy to assess the difference between phenotypes. As expected, any hyper parameter of the classifier, such as its capacity or even the way it is trained, would add its form of bias between the images and our actual understanding of what the difference between conditions might be. A classifier would also silently leverage invisible biases for discrimination. In contrast our approach highlights the signal difference that discriminates between two close phenotypes directly, without the need for an intermediate classifier.

A current limitation of conditional GAN to explain the variation between invisible phenotypes is that it cannot be used to artificially alter a real image. Instead of using a conditional GAN, an unconditional GAN along with GAN inversion could be used to obtain phenotype translation using our approach (see method 2 in online methods). However, attempting to use several GAN inversion methods to retrieve latent code from a real image in a GAN or in a conditional GAN latent representation led to unsatisfactory image results on visible cell phenotypes^25,26^. Even some high concentration perturbation images could not be reconstructed properly by swapping important details and organelle positioning, which we considered even more problematic in identifying subtle phenotypes. Many reasons can explain these results as assessed in a recent review on GAN inversion which is still an open problem^27^. On the other hand, we demonstrated here that performing transformation on real images was definitely not required in order to decipher variations of a subtle phenotype. Another apparent constraint of the proposed approach is that it still requires a human to infer the difference from the visual transformation and does not discriminate relevant from irrelevant differences on its own. While this could be seen as a limitation, it in fact indicates that the system is unbiased. Datasets do include differences and a conditional GAN will just display them without favoring relevant biological features over unwanted biases. Using such an approach, we could then reverse the classifier explanation paradigm, and rather than explaining a posteriori a classifier trained blindly, we could envision first extracting relevant and irrelevant differences between two conditions from a dataset using this approach, and then building an efficient guided system that would consider only the features of interest, disregarding the identified biases or irrelevant features. The staining artifacts in the red blood cell example is typically such an irrelevant feature that we would rather not want a classifier upon which to base its discrimination.

An extension of this work could consider disentangling the image transformation into interpretable factors of variation so that the weight of quantitative features may be better established and used subsequently. Furthermore, while we demonstrated that the proposed method can translate a cell population from a condition to another, we cannot strictly make this claim at the single-cell level because the training phase does not have access to the single-cell transformation information. Although it would necessitate datasets including time lapse or cell correspondence between conditions, an interesting extension of this method could then be used to enforce learning of single-cell trajectories during training. This would make it possible to infer whether or not a perturbation is contingent on initial cell state.

This method is straightforward to use, can be applied to any microscopy image modality and is accessible to the community. Beyond displaying invisible phenotypic variations, this system proposes a straightforward way to discover yet unknown biomarkers.

## Methods

### Ethical statement

Our research complies with all relevant ethical regulations for the boards/committees and institutions that approved the study protocols. The study on dopaminergic neurons only used existing, cultured iPSC lines deposited in the European Bank for Induced Pluripotent Stem Cells (EBiSC, https://cells.ebisc.org/) and listed in the Human Pluripotent Stem Cell Registry (hPSCreg, https://hpscreg.eu/). Post-mitotic neurons from those iPSCs were obtained by a commercial provider (Life & Brain GmbH, Bonn, Germany, doi: 10.1016/j.stemcr.2022.09.001). Detailed information for the used lines is available in hPSCreg (https://hpscreg.eu/cell-line/STBCi004-B). The malaria slides come from a survey carried out in the field in Benin. Participants are people of both sexes from one year and older. An information note was presented to them in the local language and they signed an informed consent (parents signed for minors). These documents were approved by the ethics committee of the Cotonou Entomology Research Center. The study received approval from the institutional ethics committee of the Center for Research in Entomology of Cotonou n°023/CREC/CEI-CREC/SA.

### Conditional GAN

A cell image transformation between phenotypes can be obtained using a conditional GAN by producing a latent traversal of a single random seed between two trained conditions. Any GAN architecture can be made conditional by modifying the regular GAN minimax game:

1 $${\min }_{G}\,{\max }_{D}{{{{{\mathcal{V}}}}}}\left(G,\, D\right)=	{{\mathbb{E}}}_{{{{{{\bf{x}}}}}}\! \sim \!{p}_{{{\mbox{data}}}}\left({{{{{\bf{x}}}}}}\right)}\left[\log D\left({{{{{\boldsymbol{x}}}}}}\right)\right] \\ 	+{{\mathbb{E}}}_{{{{{{\bf{z}}}}}}\sim {p}_{z}\left({{{{{\bf{z}}}}}}\right)}\left[\log \left(1-D\left(G\left({{{{{\bf{z}}}}}}\right)\right)\right)\right]$$

by introducing auxiliary information both in the generator G and the discriminator D:

2 $${\min }_{G}\,{\max }_{D}\,{{{{{\mathcal{V}}}}}}(G,\,D)=	{{\mathbb{E}}}_{{{{{{\bf{x}}}}}}\!\sim \!{p}_{{{\mbox{data}}}}({{{{{\bf{x}}}}}})}\left[\log D({{{{{\bf{x}}}}}}{{{{{\rm{|}}}}}}c)\right] \\ 	+{{\mathbb{E}}}_{{{{{{\bf{z}}}}}}\!\sim \!{p}_{z}({{{{{\bf{z}}}}}})}\left[\log (1-D(G({{{{{\bf{z}}}}}}{{{{{\rm{|}}}}}}c)))\right]$$

In practice, a linear layer is used in both the generator networks G and the discriminator D to embed each condition as a learnable vector. This embedding vector is then concatenated to an early layer of the generator. Similarly, a condition embedding vector is concatenated or multiplied to the output of one of the deepest layers of the discriminator. At training time, the conditional generator is trained to fool the conditional generator for each condition. The trained generator acts as a bank of unconditional GANs but presents the advantage of sharing the same latent representations.

### Translation and latent traversal

The output of each layer of a deep network is a latent representation of the network input. Any displacement or translation in one of these latent spaces in a conditional or unconditional GAN will produce an image transformation as output. We define the phenotype translation as the unique translation that transforms the same random seed z (a synthetic image of a cell) from a condition c_1_ to a condition c_2_. Also, when considering subtle phenotypes, we found it very useful and insightful in practice to actually compute and display the latent traversal, i.e. the consecutive images showing the transformation from one phenotype to the other. While the gradual transformation dynamic is not biologically relevant, each image is a possible intermediate state because it was assessed as realistic by the discriminator. Furthermore, it allows us to gradually see the modifications of fine features that need to occur in order to transform an image from one condition to another. This is especially interesting in the case of subtle phenotypes, when conditions are very similar.

We have identified three ways to generate latent traversal between conditions that can each be used for different purposes and on different GAN architectures.

The first approach can be used with any conditional GAN architecture and simply consists of a linear interpolation between the embedding vectors of two conditions for the same random seed. Given a generator G that includes an embedding layer E that maps a condition c to a vector e and the remaining layers F that takes a random seed z and an embedding vector e, then G(z,c) = F(z,E(c)). A latent traversal of N + 1 images can then be computed from the output of the embedding layer in this way:

3 $$	{T}_{G}({{{{{\bf{z}}}}}},\,{c}_{1},\,{c}_{2})={T}_{F,E}({{{{{\bf{z}}}}}},\,{c}_{1},\,{c}_{2})=\\ 	\left\{F\left({{{{{\bf{z}}}}}},\frac{i}{N}E({c}_{1})+\left(1-\frac{i}{N}\right)E({c}_{2})\right),\, i\in \{0,..,N\}\right\}$$

The second approach is specific to StyleGAN2, an efficient architecture used here that builds an intermediate latent representation named W that, by construction, tends to be disentangled. It is then possible to directly interpolate in the W space, which is interesting as it also allows us to compute latent traversals without prior annotations, which is useful if one wishes to modify real unannotated image after GAN inversion, compute distances to obtain dose responses curves, or perform projections, as it is done in the manuscript. StyleGAN2 is composed of a mapping network M and a synthesis network S. A random seed z and a condition c are combined to obtain the intermediate representation w = M(z,c), then an image can be synthesized with S(w). The centroid of the distribution of the intermediate representations of a treatment c_j_ can be estimated by simply computing the sample mean of K (e.g. 100,000) random seeds:

4 $${\bar{{{{{{\bf{w}}}}}}}}_{j}=\frac{1}{K}\mathop{\sum }\limits_{k=1}^{K}M({{{{{{\bf{z}}}}}}}_{k},\, {c}_{j})$$

Applying the distribution shift from c_1_ to c_2_ to a given seed z can then be computed in this way:

5 $$	{T}_{S,M}({{{{{\bf{z}}}}}},\,{c}_{1},\,{c}_{2})=\\ 	\left\{S\left(\frac{i}{N}M({{{{{\bf{z}}}}}},\,{c}_{1})+\left(1-\frac{i}{N}\right)\left[M({{{{{\bf{z}}}}}},\,{c}_{1})+({\bar{{{{{{\bf{w}}}}}}}}_{2}-{\bar{{{{{{\boldsymbol{w}}}}}}}}_{1})\right]\right),\, i\in \{0,..,N\}\right\}$$

This approach can also be used with unconditional GANs to find axes of variations by inverting sets of images rather than synthesizing them.

The third approach is also specific to StyleGAN2 and can only work with generated images. A latent traversal corresponding to a phenotype translation can be obtained in a straightforward way by considering linear interpolation between the locations of the same random seed z conditioned by c_1_ and c_2_ in W:

6 $${T}_{S,M}({{{{{\bf{z}}}}}},\,{c}_{1},\,{c}_{2})=\left\{S\left(\right.M\left(\frac{i}{N}M({{{{{\bf{z}}}}}},\,{c}_{1})+\left(1-\frac{i}{N}\right)M({{{{{\bf{z}}}}}},\,{c}_{2})\right),\,i\in \{0,..,N\}\right\}$$

Note that these 3 approaches are implemented and available in our Github repository and led in practice to very close results; we thus mostly used the third approach in this work since for the sake of explanation we only need to manipulate synthetic images, and we did choose to use StyleGAN2 which is currently considered one of the best GAN by the computer science community.

### Conditional GAN training and inference

We developed scripts to prepare any image dataset with two or more conditions for a conditional StyleGAN2 training and for performing image translation through latent interpolation (https://github.com/biocompibens/phenexplain). Once the dataset was prepared with each image conditionally annotated and packed into a single zip file, training for each dataset was performed on a Computing server equipped with 4 NVidia A100 GPU which took between 6 h and 48 h depending on the dataset. BBBC021 took the longest time because it is made of 73 conditions (45 million images were seen in 48 h). We stopped training when FID plateaued long enough (~5 million images). Our translation script generates latent traversal videos of a grid of any number of samples (in row) from any condition to one or several other conditions (columns). The StyleGAN2 and Frechet Inception Distances (FID) implementations used in this paper are available from https://github.com/NVlabs/stylegan2-ada-pytorch. Refer to our github (https://github.com/biocompibens/phenexplain) to perform cell image translation with the models trained for this paper or to train and use this method on another dataset.

### Frechet inception distance

The Frechet Inception Distance measures the difference between the Inception representations of two image sample distributions. While the use of FID is debated in the literature, there is no ideal metric for assessing synthetic image quality and FID is still the most widely accepted reference to evaluate the capability of a generative model to reproduce an image distribution: a low FID value means the distribution of real image is close to the distribution of generated images in the inception representation (in the Frechet distance sense). We provide FID values for the conditional generative models we trained on each dataset and FID values in comparison to real natural images for comparison (Supplementary Table 2**)**. We also performed an experiment where we gradually corrupted cell images to provide a scale and a threshold at which FID can still be considered as good in this context (Supplementary Fig. 7).

### Convolutional Neural Networks classification

Convolutional Neural Networks (CNNs) were employed to provide evidence that the various visually indiscernible conditions used in this paper were discernible by a deep network (Supplementary Table 1). In this case, as we had large datasets with annotated conditions, the network was kept as simple as possible, made up of only four convolutional layers and trained for 10 epochs on each dataset using a 4-fold cross-validation. The mean and standard deviation of the accuracy over these 4 runs were then reported. CNNs were also used to quantitatively validate on real images the intuitions provided by conditionally generated images (Supplementary Figs. 12 and 14). In this case, as we had a large dataset but a limited amount of manually annotated image phenotypes, we took advantage of transfer learning and used a ResNet50V2 model pre-trained on ImageNet. We then fine-tuned the model on our datasets based on small (~200) annotated cell image sets (hole vs other, crenated vs other, neurons vs non neurones, long neurite vs short neurites).

### Phenexplain web interface

For ease of understanding and visualization, we created an interactive web interface with data produced from all the datasets we used in this study, making it possible to explore and manipulate more examples than the one presented in the figures (https://www.phenexplain.bio.ens.psl.eu/). For all datasets, latent traversal can be explored interactively for a selection of examples. For the BBBC021 dataset, which provides more conditions, a more detailed exploration is possible. First, a linear discriminant analysis (LDA) was used to provide a projection of the latent space that properly separates three conditions (DMSO, and nocodazole or cytochalasin B at a high concentration). Their respective regions in the latent space can be explored to show that a variety of images can be generated for each condition. Latent traversal from DMSO to a compound can be performed either through a linear interpolation between DMSO and the highest concentration, or by successive interpolations between intermediate concentrations. We expect the latter to provide a more accurate trajectory, but the projection of these points onto the linear interpolation shows that the former is a reasonable approximation.

### Golgi assay

HeLa cells obtained from the commercial provider American Type Tissue Collection (ATCC Cat# CCL-2, RRID:CVCL_0030) stably expressing EGFP-CCR5 (CC chemokine receptor 5) were kindly provided by F. Perez’s team, UMR144, Institut Curie) and cultured as described in ref. ^28^. Cells were briefly grown in Dulbecco’s modified Eagle’s medium (DMEM) (Thermo Fisher Scientific) supplemented with 10% fetal calf serum (FCS), 1 mM sodium pyruvate, and penicillin and streptomycin (100 μg/ml) (Thermo Fisher Scientific). For the Golgi assay, 5.0 × 10^3^ cells were seeded on black clear-bottom 384-well plates (ViewPlate-384 Black, 784201, PerkinElmer) in 40 μl of complete medium. Twenty-four hours after cell seeding, DMSO (control solvent) and nocodazole were transferred robotically to plates containing cells to a final concentration of 10 μM and 0.5% of DMSO. After 90 min of incubation, cells were treated with 40 μM biotin for 120 min at 37 °C. Cells were processed immediately after biotin treatment for immunofluorescence. Briefly, cells were fixed with 3% paraformaldehyde for 15 min and quenched with 50 mM NH_4_Cl in phosphate-buffered saline (PBS) solution for 10 min. Nuclei were counterstained with DAPI (D3571, Life Technologies, Introvigen) at dilution 1:500 for 45 min. Image acquisition was performed using an INCell 2200 automated high-content screening fluorescence microscope (GE Healthcare) at a ×20 magnification (Nikon 20×/0.45). Four image fields were acquired per wavelength, well, and replicate experiment.

### NF-κB nuclear translocation assay

HCC1143 cancer cells were obtained from the commercial provider American Type Tissue Collection (ATCC Cat# CRL-2321, RRID:CVCL_1245) and cultured in RPMI 1640 medium (11875085, Gibco Thermofisher) supplemented with 10% bovine fetal serum (A5209502, Gibco Thermofisher), 100 U/mL penicillin and 100 µg/mL streptomycin in a humidified environment consisting of 95% air and 5% CO_2_ at 37 °C. Cells were seeded on black clear-bottom 384-well plates (ViewPlate-384 Black, 784201, PerkinElmer) in 40 μl of complete medium at density of 1000 cells/well for 24 h before exposure to 20 ng/ml TNFα for 30 min. After the incubation period, cells were fixed with 4% (vol/vol) buffered paraformaldehyde solution (PFA, Sigma-Aldrich) for 15 min, quenched with 50 nM NH_4_Cl solution, and permeabilized with 0.5% (vol/vol) Triton X-100 in PBS for 5 min. Cells were stained with Rabbit anti-NF-κB p65 (C-20) (Santa Cruz Biotechnology Cat# sc-372, RRID:AB_632037) at 1:200 dilution for 1 h, washed in PBS and then incubated with anti-rabbit A488 secondary antibodies (Molecular Probes Cat# A-21206, RRID:AB_2535792) at 1:1000 dilution together with 4’,6-diamidino-2-phenylindole dilactate (DAPI D3571, Invitrogen) at dilution 1:500 for labeling nuclei. Image acquisition was performed using an INCell 2200 automated high-content screening fluorescence microscope (GE Healthcare) at a ×20 magnification (Nikon 20×/0.45). Four image fields were acquired per wavelength, well, and replicate experiment.

### Isogenic iPSC lines

The iPSC lines used in this paper were generated by a third party and are described in detail in^29^. The iPSC lines are deposited in the European Bank for Induced Pluripotent Stem Cells (EBiSC, https://cells.ebisc.org/) and listed in the Human Pluripotent Stem Cell Registry (hPSCreg, https://hpscreg.eu/). Detailed information for the used lines is available in hPSCreg (https://hpscreg.eu/cell-line/STBCi004-B). iPSCs were cultivated on Geltrex-coated (Thermo Fisher Scientific) dishes in StemMACS iPS-Brew XF (Miltenyi Biotech). The medium was changed daily, and cells were passaged twice a week using 0.5 mM EDTA in PBS (Thermo Fisher Scientific). Mycoplasma testing was performed twice per month.

### Differentiation of dopaminergic neurons from iPSCs, immunohistochemistry and image acquisition

Dopaminergic neurons were differentiated from iPSCs using a modified protocol based on Kriks et al. which is described in more detail in Vuidel et al. (Kriks et al., 2011; Ryan et al., 2013; Weykopf et al., 2019; Vuidel et al., 2022). Differentiated dopaminergic neurons were cryopreserved at 30 days in vitro (DIV30). For further experimentation, cryopreserved neurons were thawed in a water bath and centrifuged (400 g, 5 min, RT) in basal medium supplemented with ROCK inhibitor (Miltenyi, #130-095-563). Cell pellets were resuspended in differentiation medium supplemented with a ROCK inhibitor. Media compositions are detailed in Vuidel et al., 2022. 384-well plates (Perkin Elmer, #6007558) were coated with 15 μg/ml Poly-L-Ornithin for 1 h at 37 °C followed by 10 μg/ml Laminin overnight at 4 °C. Using Tryphan Blue (Sigma, # T8154-20ML) and a Countess automated cell counter (Invitrogen), 10,000 cells/well were seeded in 384-well plates. Edge wells were avoided for seeding and filled with PBS. Thawed cells were incubated at 37 °C and 5 % CO2 for seven days until 37 DIV with differentiation medium changes every other day. Cell fixation and immunohistochemistry were performed as detailed in Vuidel et al., 2022. Neural cell assay was stained with Rabit anti-tyrosine hydroxylase (https://www.merckmillipore.com/KR/en/product/Anti-Tyrosine-Hydroxylase-Antibody,MM_NF-AB152) at dilution 1:1500, a marker for dopaminergic neurons and Mouse anti-α-Synuclein (https://www.bdbiosciences.com/en-us/products/reagents/microscopy-imaging-reagents/immunofluorescence-reagents/purified-mouse-anti-synuclein.610787) at dilution 1:500 that accumulates in Lewy bodies and Lewy neurites in Parkinson’s disease. For secondary antibodies, Anti Rabbit - Alexa Fluor 488 (Goat Anti Rabbit–Alexa fluor 488: A-11008 Life Technologies) at dilution 1:1000 and anti-mouse-Alexa Fluor 633 (Goat anti-mouse-Alexa Fluor 633: A-21050 Life Technologies) at dilution1:1000. There was no mycoplasma contamination. All imaging experiments were performed on a Yokogawa CV7000 microscope in scanning confocal mode using a dual Nipkow disk. 384-well plates (Perkin Elmer, #6007558) were mounted on a motorized stage and images were acquired in a row-wise “zig-zag” fashion at RT.

### Malaria microscopic and qPCR diagnoses

Whole blood specimens sampled on EDTA were initially collected during a population survey of asymptomatic patients for malaria infection in Benin. From each blood sample, approximately 8 µl and 2 µl of whole blood were carefully placed on a microscopic slide to respectively perform thick and thin blood film for microscopic examination. Thick blood film was stained by giemsa 8% after drying whereas thin blood film was stained with conventionnal may-grunwald-giemsa after fixation by methanol. Microscopic examination was performed by a skilled microscopist and was considered to be negative if no circulating parasites had been observed on the entire thick blood film or after microscopic screening of 40000 red blood cells on the thin blood film. If parasites were detected by microscopy, parasite load was estimated in parasites per microliter considering respectively 8000 leukocytes or 46000 red blood cells per microliter on thick and thin blood film. For the purpose of this study, a portion of the thin blood film was scanned using the AxioScan Z1 (Carl Zeiss Meditec) system at 40X and retrieved 20,000 images of size 256 × 256 pixels per slide. 300 out of 20,000 images per slide were randomly selected for deep network training. Molecular investigations were performed using RT-qPCR. Briefly, for each patient, parasite DNA was extracted from 200 µL of whole blood using DNA mini kit as recommended by the manufacturers (QIAGENⓇ) and eluted in 60 µL of buffer. Parasite DNA genomic was detected by a screening biplex RT-qPCR simultaneously targeting the 18 S gene of *P. falciparum* and *Plasmodium* spp in one well and *P. ovale* and *P. malariae* 18S gene in a second well. PCR conditions were: denaturation 10 s at 95 °C following by 40 cycles of amplification composed by denaturation at 95 °C 5 s and hybridation 1 min at 60 °C. *Plasmodium falciparum* parasite load (parasite/µL) was estimated using a standard calibration curve obtained with 3D7 *P. falciparum* strain DNA extracted from blood pellet with known parasite load.

### Statistics and reproducibility

The total amount of images per dataset is available in Supplementary Table 2 and the number of distinct samples and images for each condition is available in details in Supplementary Table 3. Distinct samples are wells from well plates except for the Malaria dataset where each considered sample is a patient thin blood smear slide. 600 images were acquired from each of these slides. A distinct sample size of 3 wells was provided with the BBBC021 dataset and is a common choice for high throughput screening assays with many conditions. For the datasets we generated we did not use a statistical method to predetermined the number of replicates or the image sample size but we chose an unusually high number of replicates (30 or above) to ensure reproducibility. Except for the Malaria and the LRRK2 datasets, image crops of 128 × 128 or 256 × 256 pixels were collected around each nucleus from all the samples. For the LRRK2 dataset, images acquired on the border of the wells were discarded. For the training of StyleGAN2, no data were excluded from the datasets prepared as described above. Image analysis tasks for evaluation were performed on full datasets, except in the following cases. The classification tasks described in Supplementary Table 1 were performed while randomly excluding a third of the images for validation. FID computations in Supplementary Table 2 were performed on random subsamples, as further described in the supplementary table caption. All microscopy images displayed in the figures were linearly scaled to 8 bits using the first and ninety-ninth percentiles of the distribution of raw image pixel values.

### Reporting summary

Further information on research design is available in the Nature Portfolio Reporting Summary linked to this article.

### Supplementary information

Supplementary information

Reporting Summary

### Source data

Source Data

## Data Availability

Synthetic images of all of our datasets can easily be manipulated here: https://www.phenexplain.bio.ens.psl.eu/. As raw data we used four original datasets we generated and a subset of the BBBC021v1 image set available from the Broad Bioimage Benchmark Collection^14,30^. All datasets used in this study are available in a Zenodo repository^31^. Source data of the figure plots are provided with this paper. Source data are provided with this paper.

## Code availability

The step by step notice to train and use a conditional GAN on image datasets of biological conditions and performing latent traversal is available as Python/PyTorch scripts from our github repository (https://github.com/biocompibens/phenexplain/). The version used in this paper can be found here^32^.
