# Supplementary material for: Revealing invisible cell phenotypes with conditional generative modeling
Source: Nat Commun. 2023 Oct 11;14:6386. doi: 10.1038/s41467-023-42124-6 (PMC10567685; doi:10.1038/s41467-023-42124-6)
Supplement: Supplementary file 1 — Supplementary information [file 41467_2023_42124_MOESM1_ESM.pdf]

# Supplementary Information

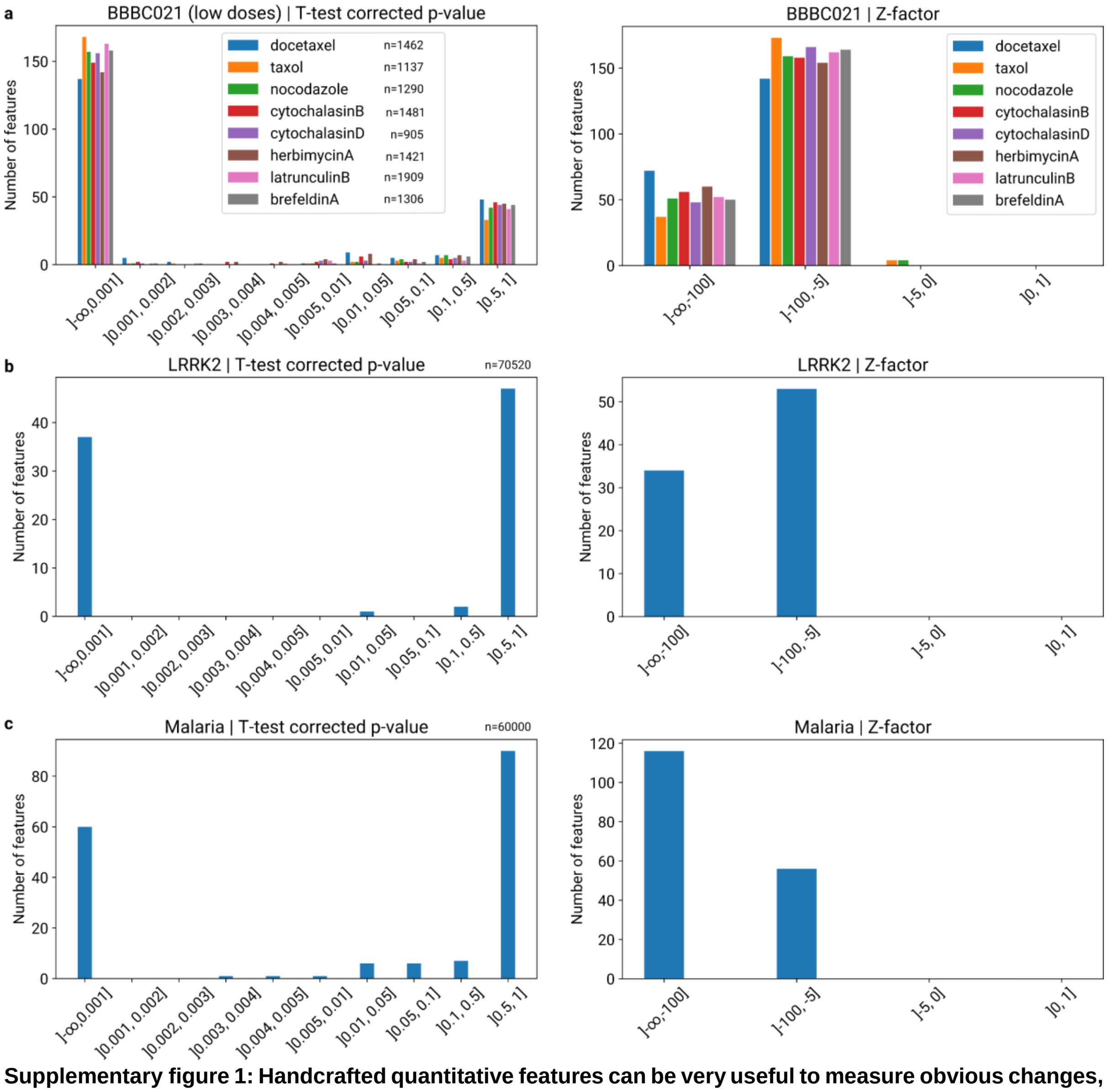


**Supplementary figure 1: Handcrafted quantitative features can be very useful to measure obvious changes, but they do not provide an intuitive solution in case of subtle phenotypic variation.** To illustrate this, we computed ~200 Cell profiler features on our 3 datasets with invisible phenotypic differences described more in depth later in the paper. **a -** BBBC021 (low doses), **b -** LRRK2 mutation and **c -**Malaria. Left plots display the distribution of p-values obtained by performing a t-test on each feature between the negative control (DMSO) and each compound at low dose for the BBC021, WT and mutation LRRK2-G2019S and Negative and Positive for Malaria. Right plots display the distribution of Z'factor for the same groups (Z' factor around 0.5 indicates a screenable effect size and a Z'factor below zero is considered not screenable). Overall, while one to three thirds of the features show a significant difference between conditions, all of them display a very low to no effect size, making a feature selection and therefore a clear interpretation difficult. For illustration, the next three figures display all the feature distributions for the two conditions in the Malaria assay (last row) to show how close distributions are in practice in the case of a subtle phenotypic change.

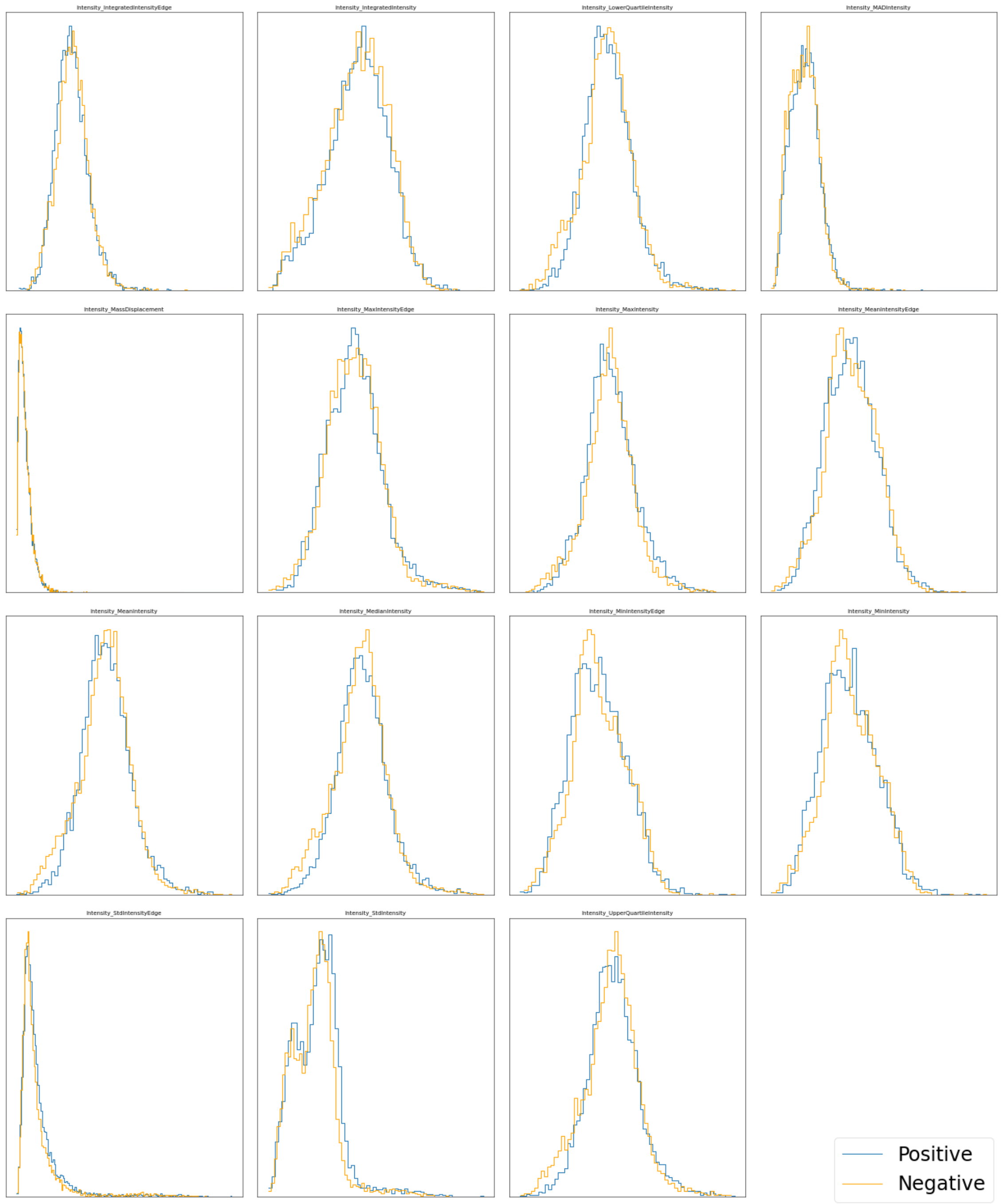


**Supplementary figure 2: Intensity features distributions computed with Cell Profiler on single cells of the malaria image dataset**. These plots and the following two supplementary figures (area and texture features) are displayed to illustrate that in case of subtle differences between conditions, common handcrafted features distributions are very close with a very low to no effect size (individual feature names for this illustration are irrelevant).

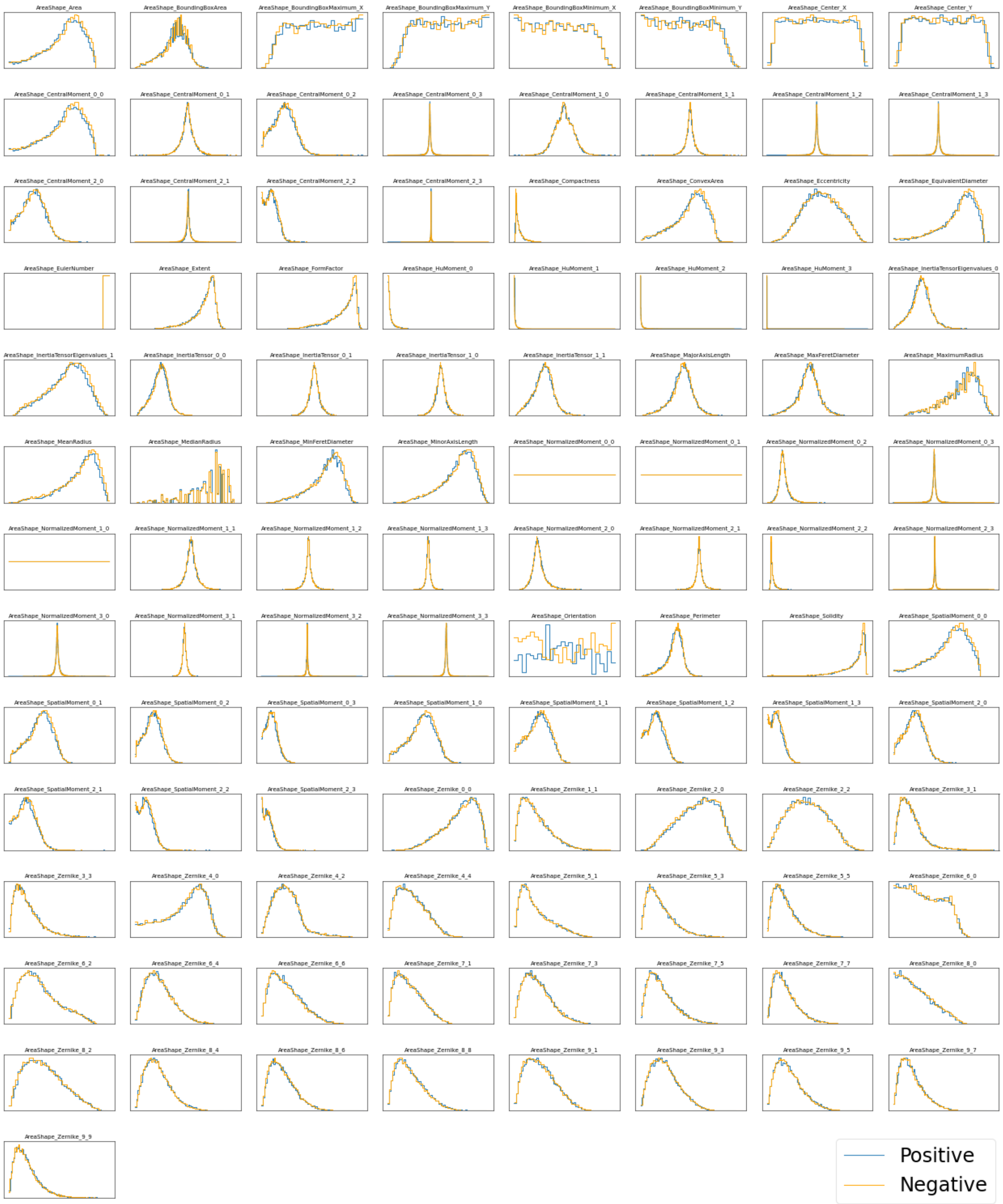


**Supplementary figure 3: Area features distributions computed with Cell Profiler on the malaria dataset.** Both conditions on all features show very close distributions (individual feature names for this illustration are irrelevant).

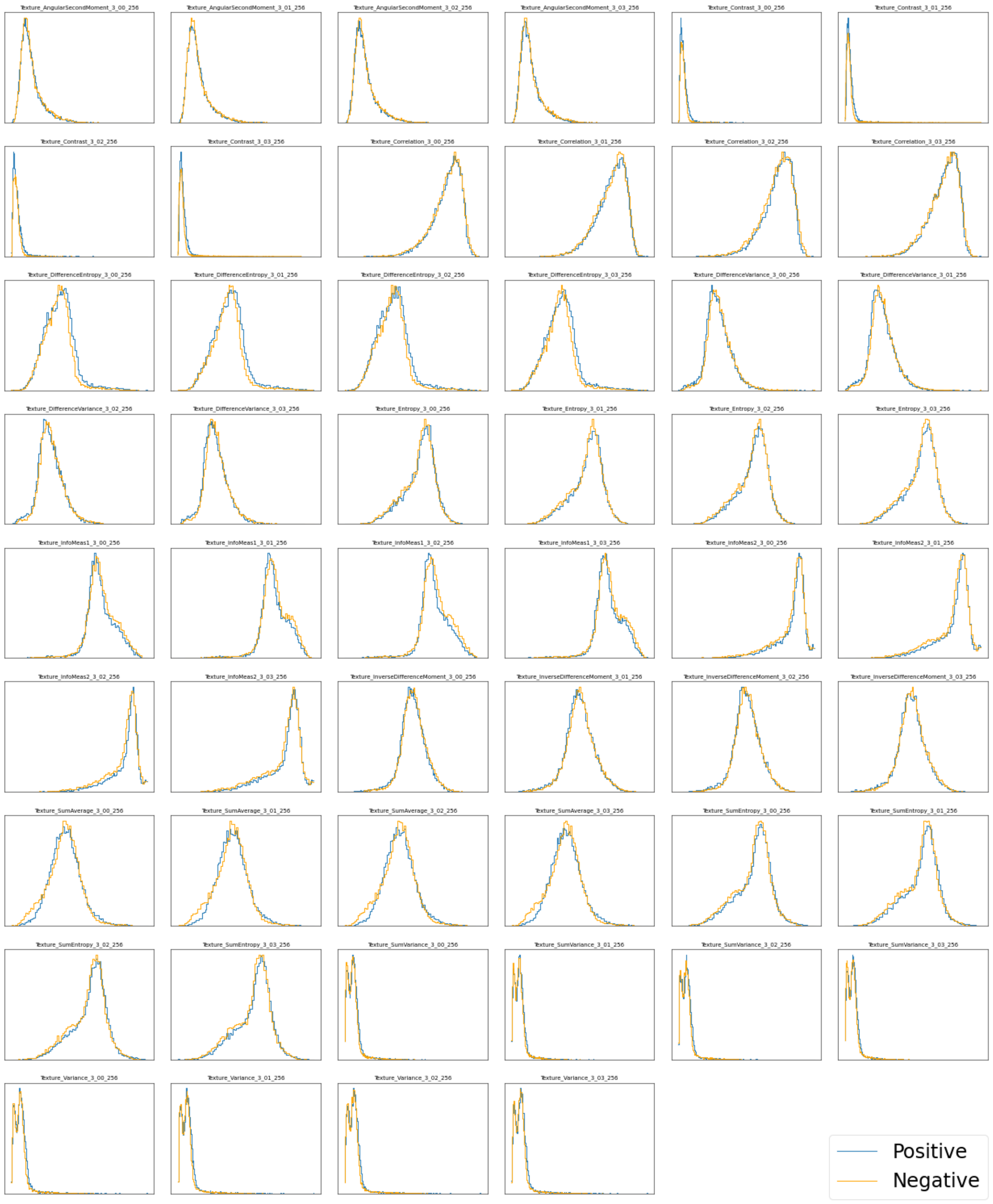


**Supplementary figure 4: Texture features distribution computed with Cell Profiler on the malaria dataset**. Both conditions on all features show very close distributions (individual feature names for this illustration are irrelevant).

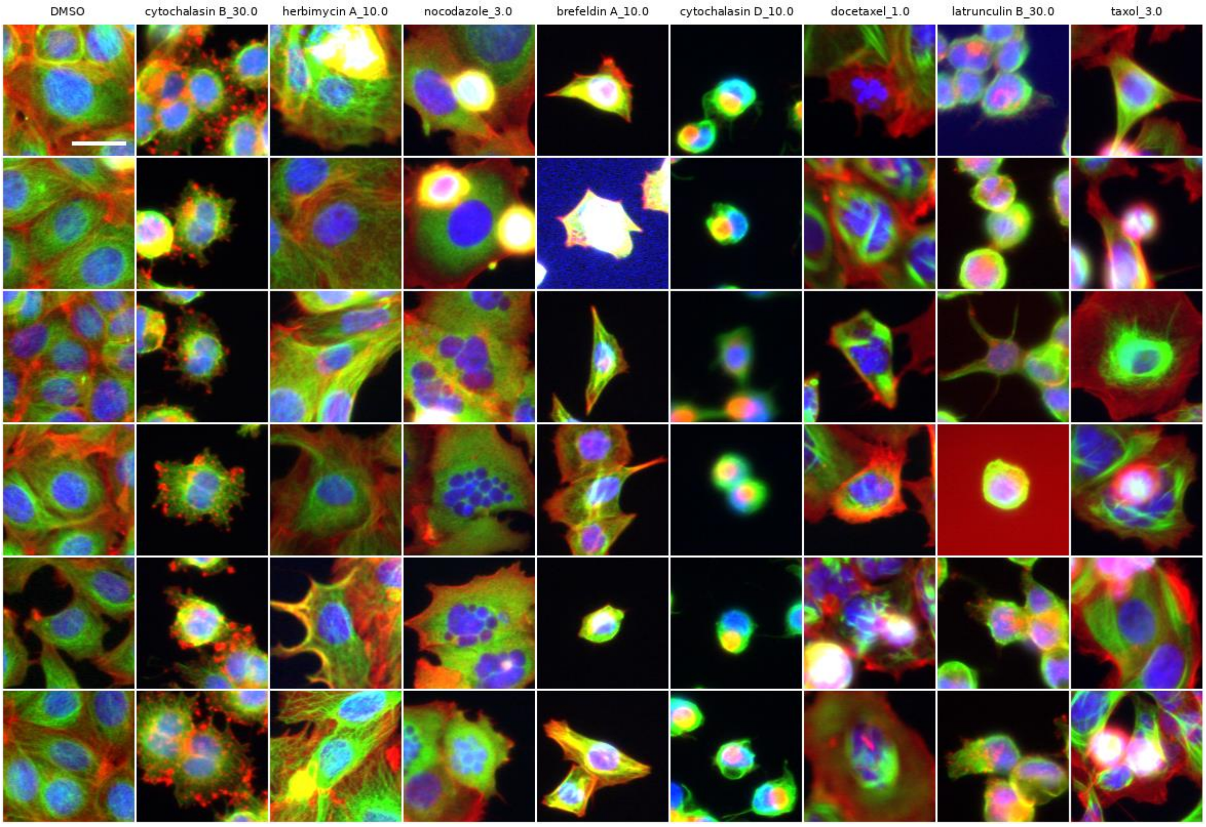


**Supplementary figure 5: Random samples (rows) of real cropped images of cells from high concentration compound treatments (columns) from the BBBC021 dataset (concentration unit is µM)**. These strong phenotypes in columns can visually be easily distinguished from one another. Scale bar is 20µm.

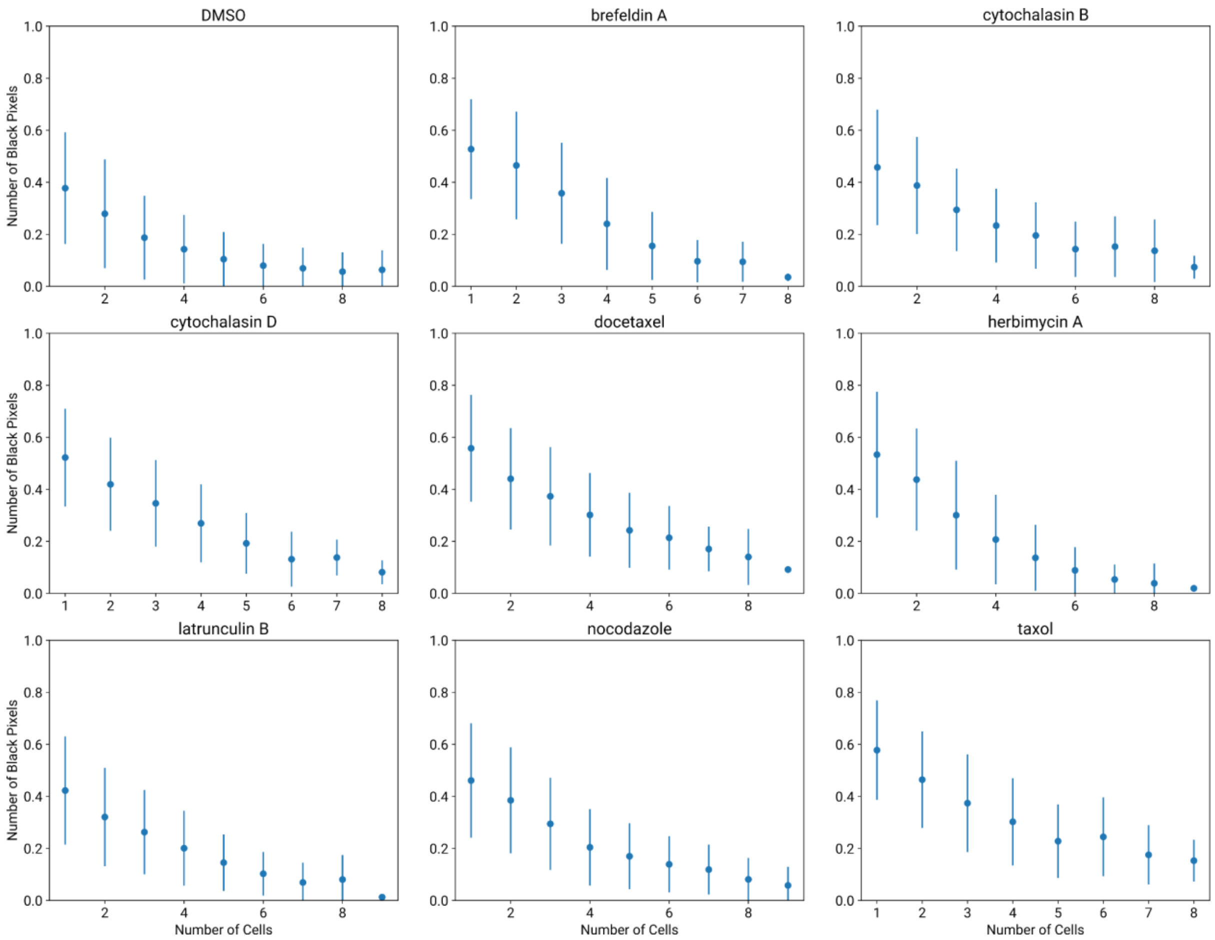


**Supplementary figure 6: The increased fraction of background in images correlates with cell count.** The fraction of image background is a direct assessment of toxicity, even at low concentration when it is barely visible on real images. Here we consider all concentrations of each of the 9 compound treatments and plot the cell count on each image used for training against real background fraction. Values are mean +/- standard deviation.

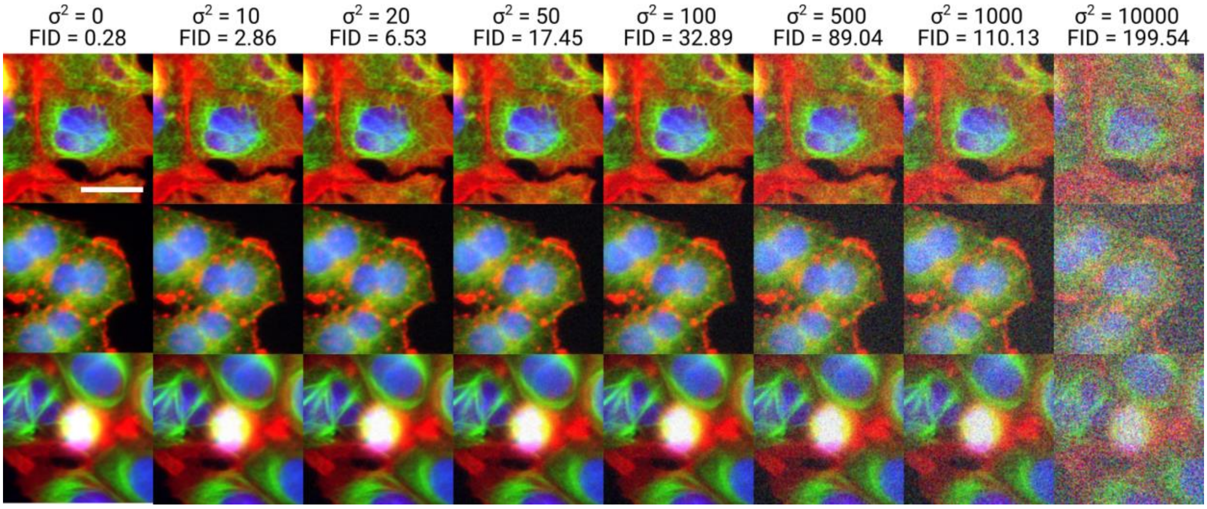


**Supplementary figure 7: Relationship between cell image degradation and FID.** For each column of this grid, an increasingly high Gaussian noise was added to one half of the BBBC021 dataset (~60k 8 bits/3 channels images 0-255; examples are displayed in rows here) to simulate increasingly degraded images. The FID was then computed between this one half and the remaining untouched half of the image dataset (~60k images). The FID computed this way for various intensities of added noise provides a scale for FID on a cell image dataset. One can see that up to at least a FID of 32, the image degradation remains invisible. Note that all of the FIDs we obtained with the trained conditional GAN in this work are below 7 (see **Supplementary Table 2**). Scale bar is 20µm.

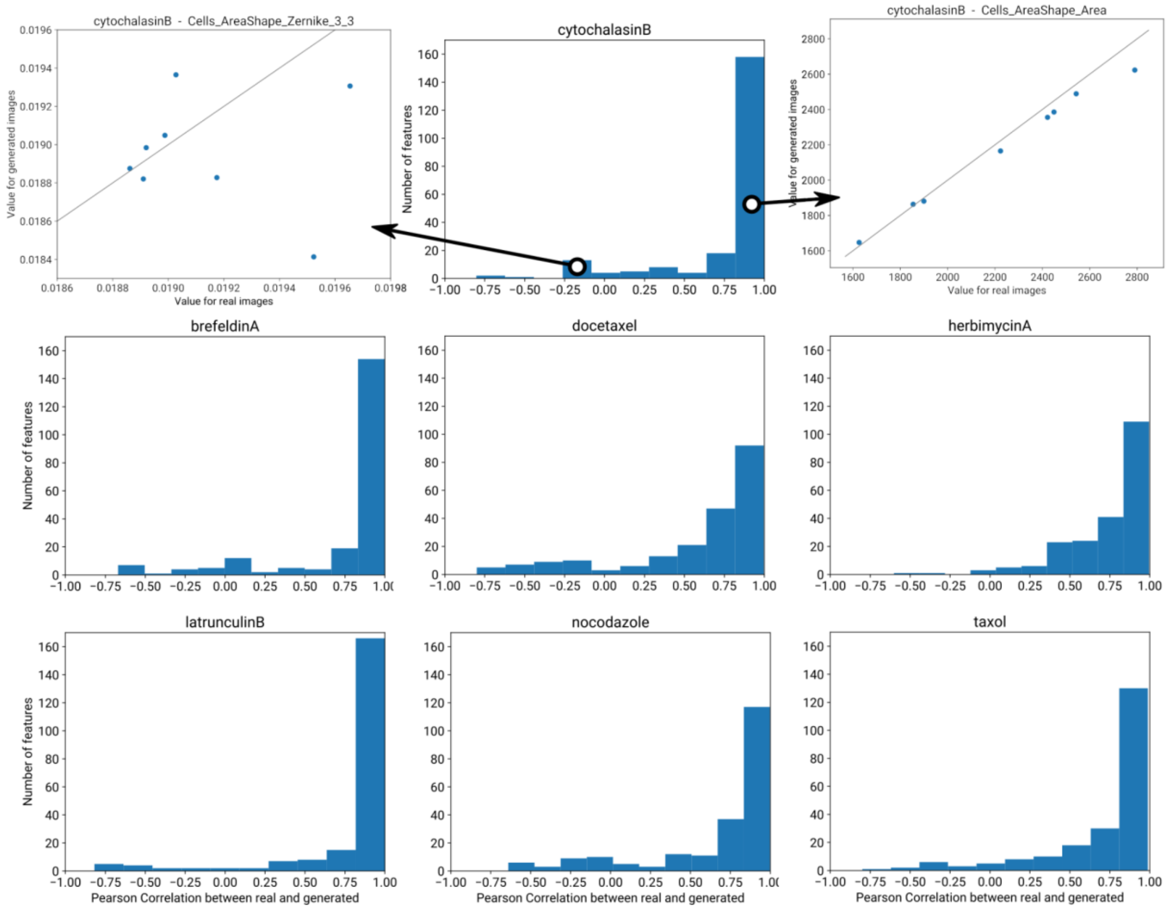


**Supplementary figure 8: Features computed from synthetic images recapitulated well features computed from real images.** We computed 213 cell profiler features for each concentration of 7 compound treatments on the real and on the synthetic images. We then computed the Pearson correlation between real and generated images for each of these 213 features and displayed the histograms obtained for each treatment. These show that most features correlate very well between real and generated pictures with a clear peak near 1. For Cytochalasin B, we show one example of features that do not correlate very well (on the left), and another example of a feature that correlates well (on the right). There was no clear rationale as to why a few features did not correlate well because they always matched hard to interpret texture features.

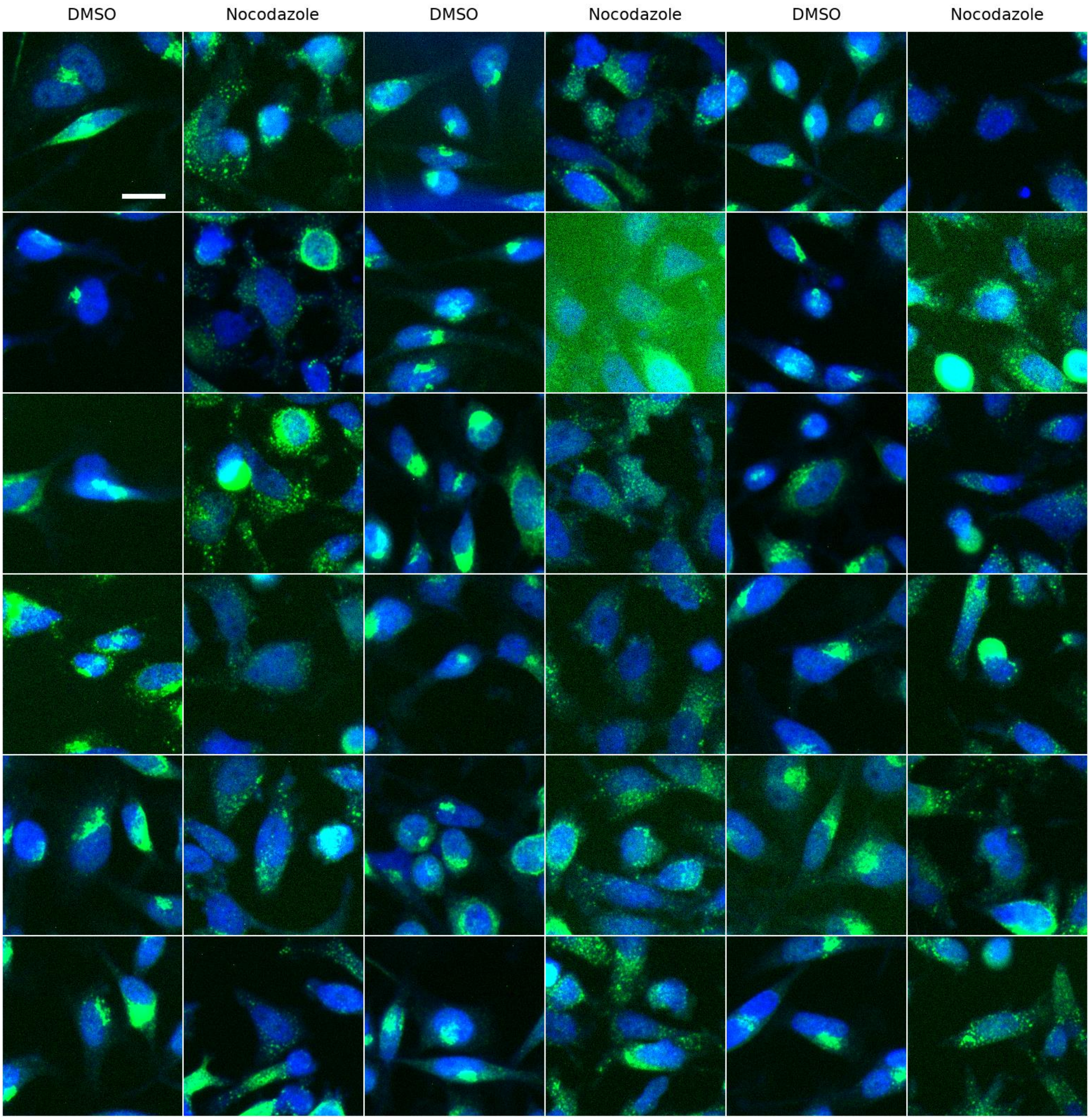


**Supplementary figure 9: Random samples (rows) of real cropped images of cells from the Golgi assay in two conditions (columns).** Nocodazole concentration is 10µM. Scale bar is 20µm.

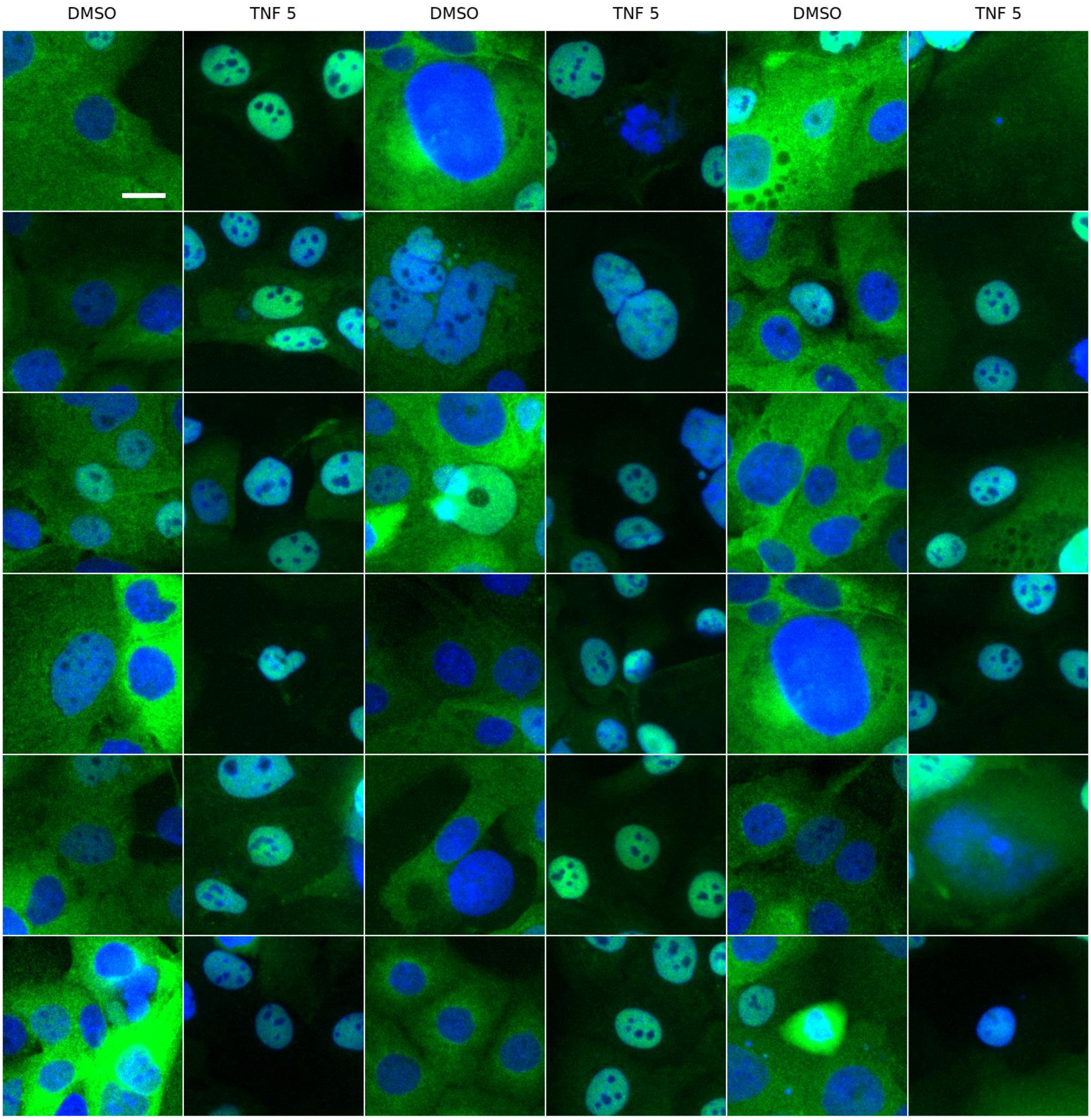


**Supplementary figure 10: Random samples (rows) of real cropped images of cells from the nuclear translocation assay in two conditions (columns).** Concentration is 5 µM. Scale bar is 20µm.

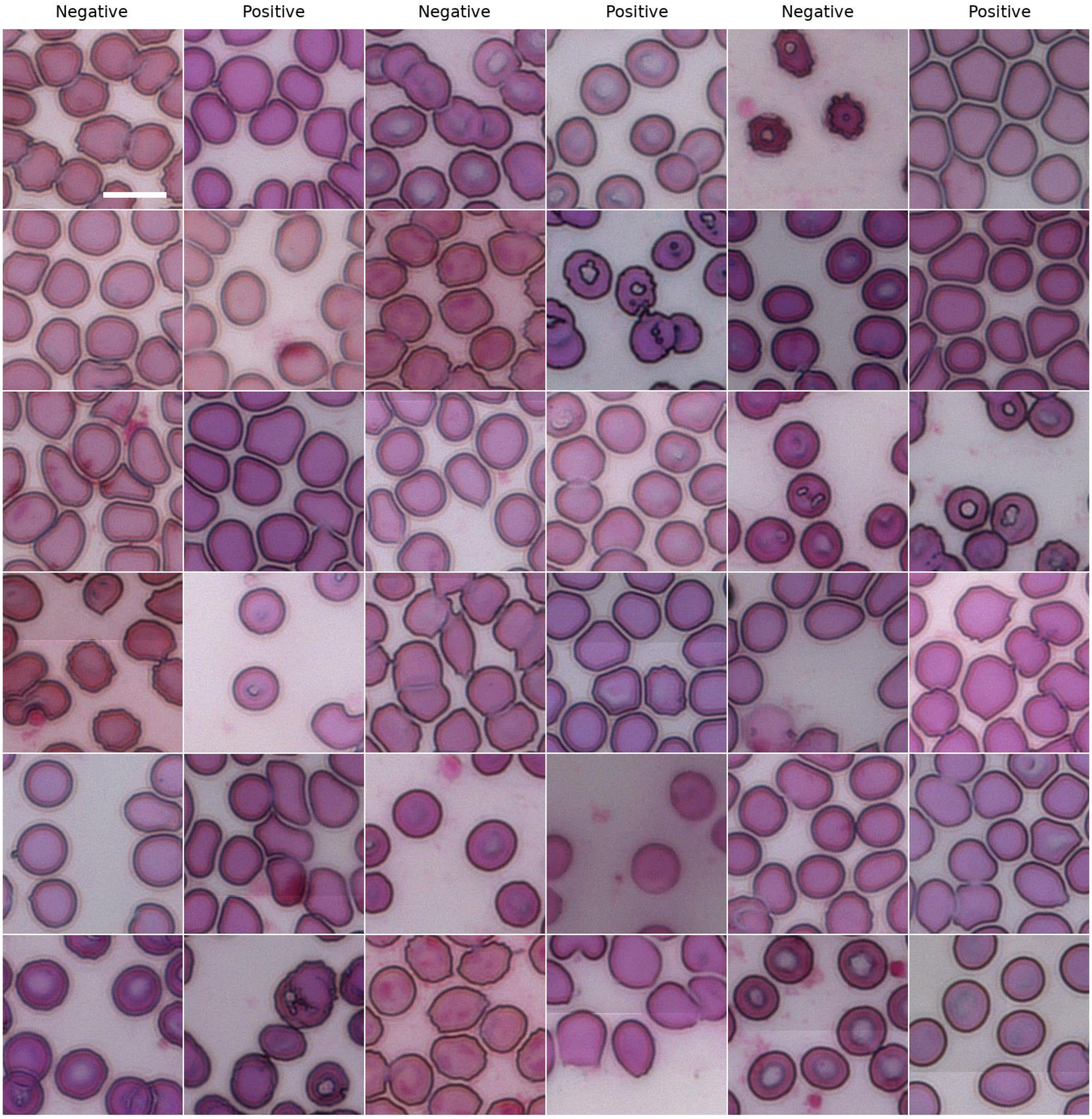


**Supplementary figure 11: Random samples (row) of real cropped images of cells from thin blood smears**. Each column displays samples of cells from negative or positive patients to a qPCR test against Malaria, but assessed as negative by a microscopist, that is without visible parasites. Columns (=conditions) cannot be visually distinguished from one another. Scale bar is 10μm.

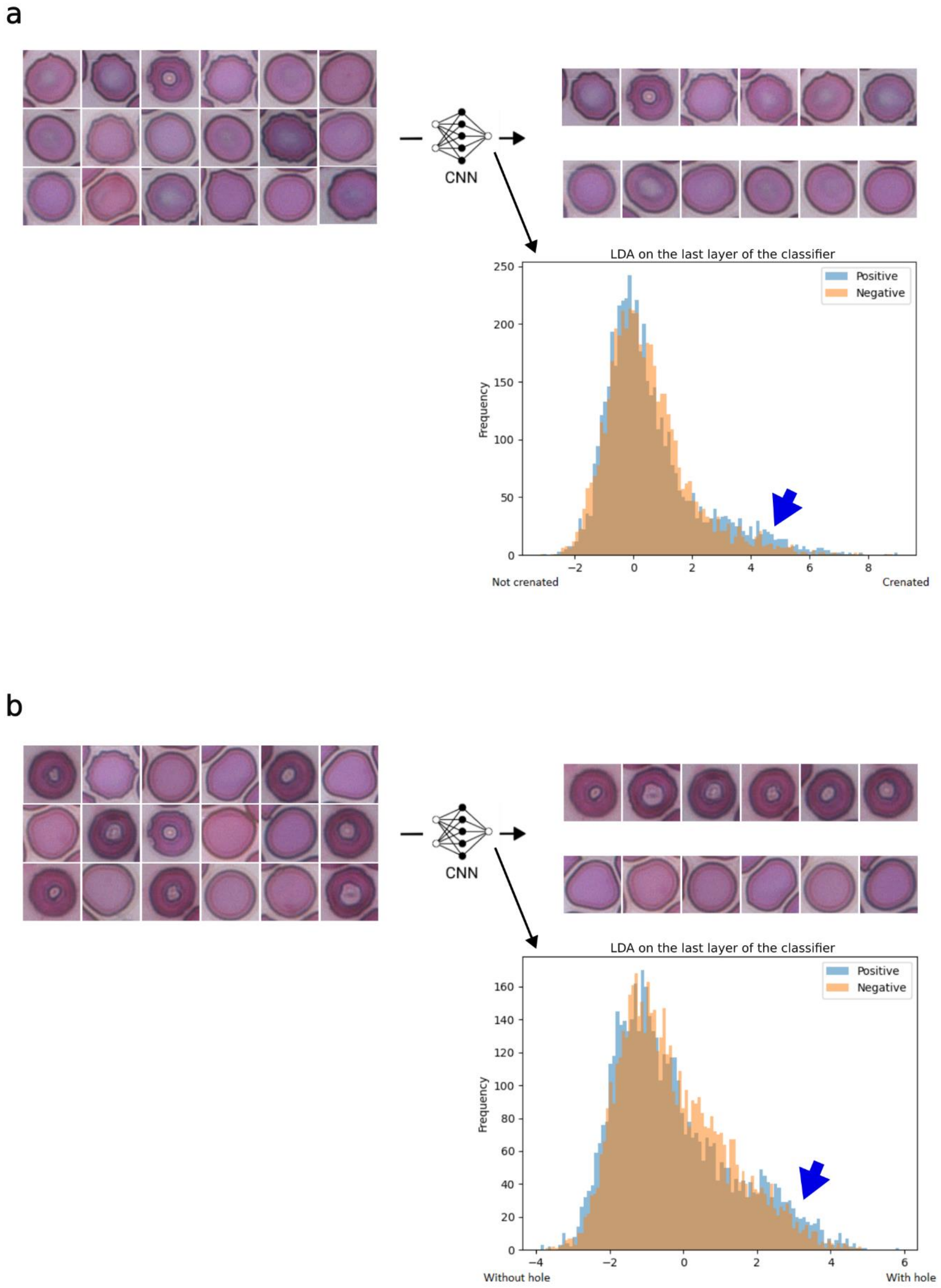


**Supplementary figure 12: Quantitative confirmation on real images of the Malaria datasets, of the intuitive observations made using our approach. a** - We trained a CNN to classify cells with a crenated border (picked up by hand) against all the other cells. We then compute a Linear Discriminant Analysis (LDA) discriminating training data from the last layer output of the CNN. We then projected the cells from negative qPCR and positive qPCR patients. The distribution confirms a slight enrichment of more crenated cells in the positive case. **b** - Same as A but for the Hole phenotype (also through a CNN trained on hand picked cells). Image crop around each red blood cell is 64x64px, 7.1µm wide.

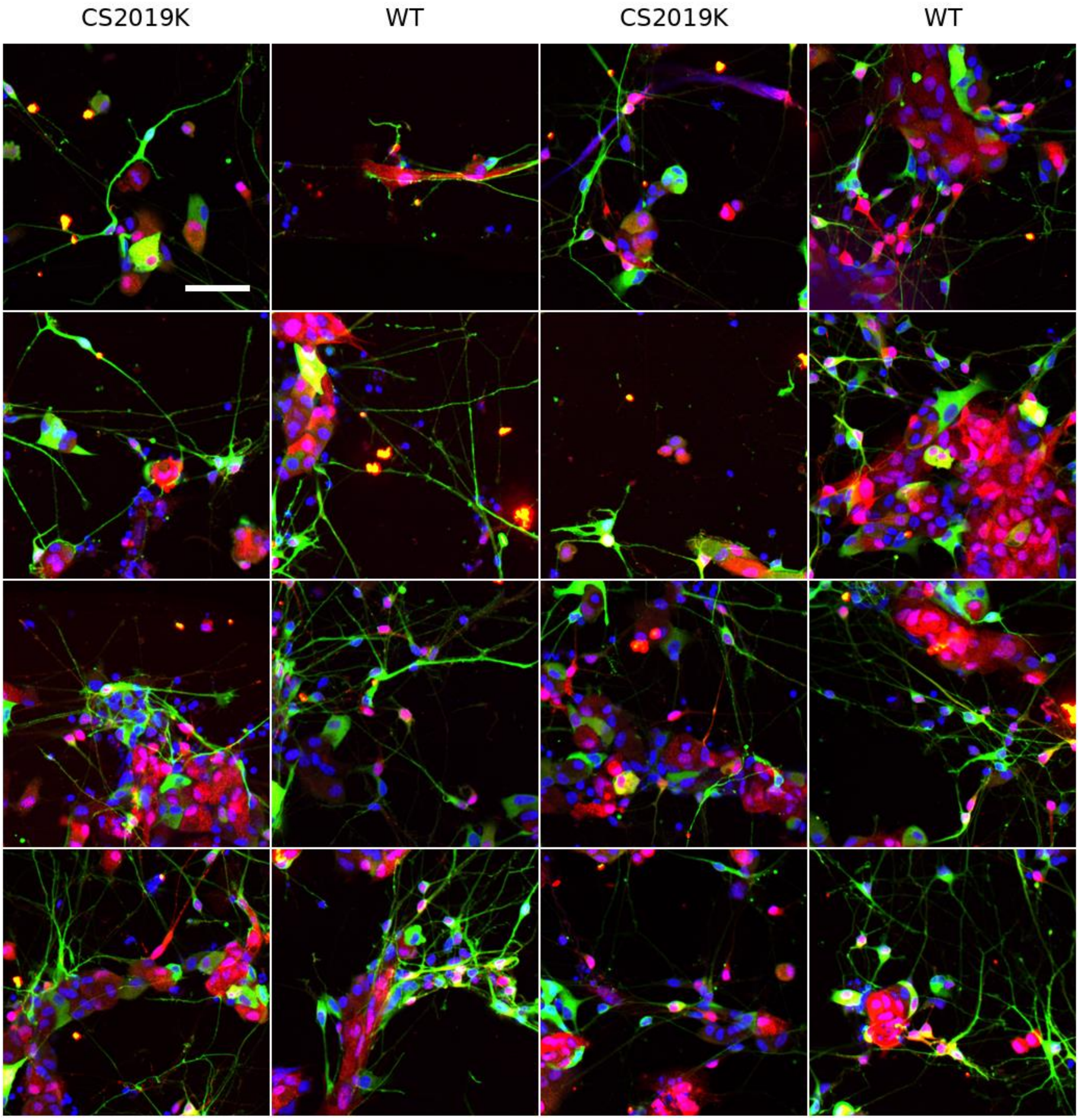


**Supplementary figure 13: Random samples (rows) of real cropped images from the two condition WT/mutant (columns) of the LRRK2-G2019S assay**. Columns (=conditions) cannot be visually distinguished from one another. Scale bar is 20µm.

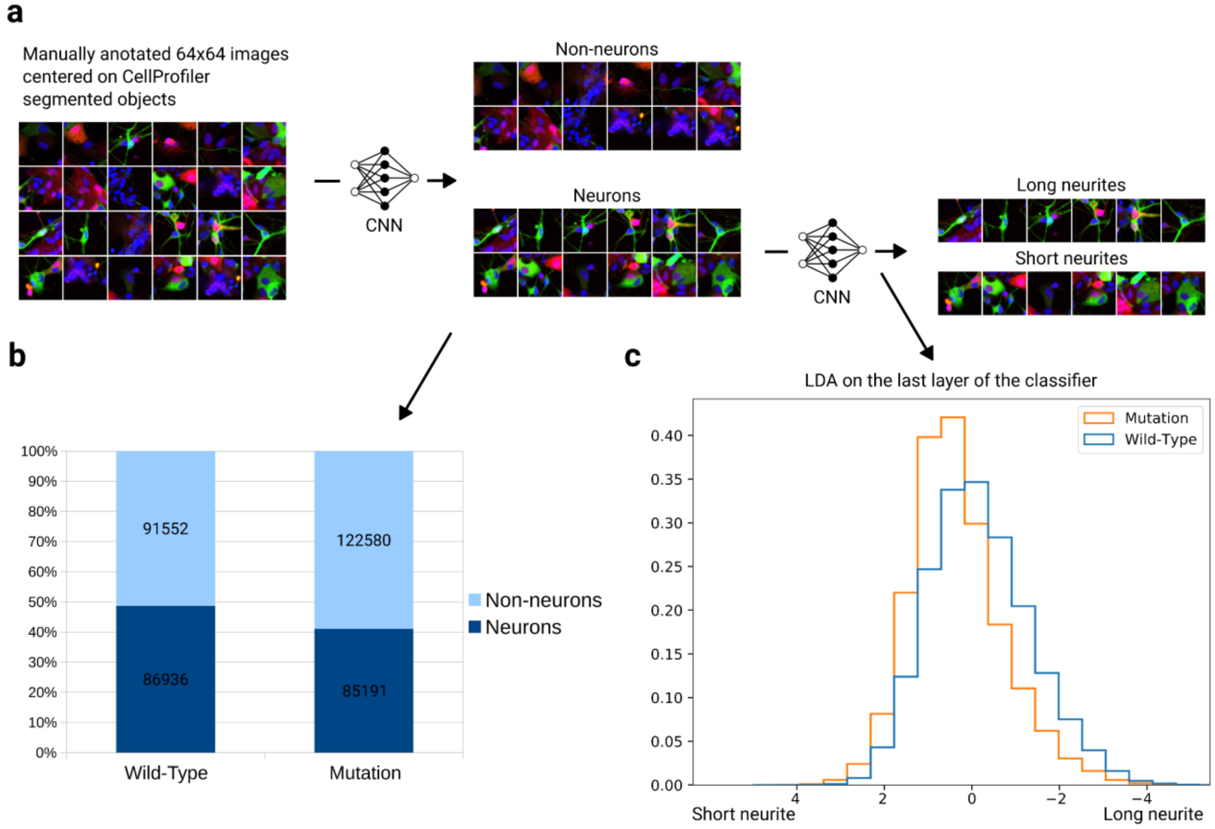


**Supplementary figure 14: Quantitative confirmation on real images of LRRK2, of the intuitive observations made using our approach. a -** We segmented nuclei using CellProfiler, but segmenting the body of individual neurons was impossible. Therefore, in order to distinguish neurons from other cells, we trained a small convolutional neural network classifier on a few manually annotated images. Then, keeping only the cells classified as neurons, we built a second classifier to learn a combination of features separating long neurites from short neurites. Importantly, these two classifiers have not seen the condition labels at training time (WT vs mutation): they can just discriminate non neuron vs neuron, and within the neurons, can measure the relative neurite length **b -** The first classifier enabled us to obtain the ratio of neurons to non-neurons in the Wild-Type and Mutation cases, showing that the Wild-Type condition contains a slightly higher percentage of neurons (indicating perhaps a difference in differentiation efficiency or cell death in this specific assay). **c -** By performing an LDA on the last layer before classification in the “neurite classifier”, we could extract a short-neurite/long-neurite axis and confirm that the Wild-Type neurons also contained longer neurites. Image crop around each cell is 64x64px, 15.2µm wide.

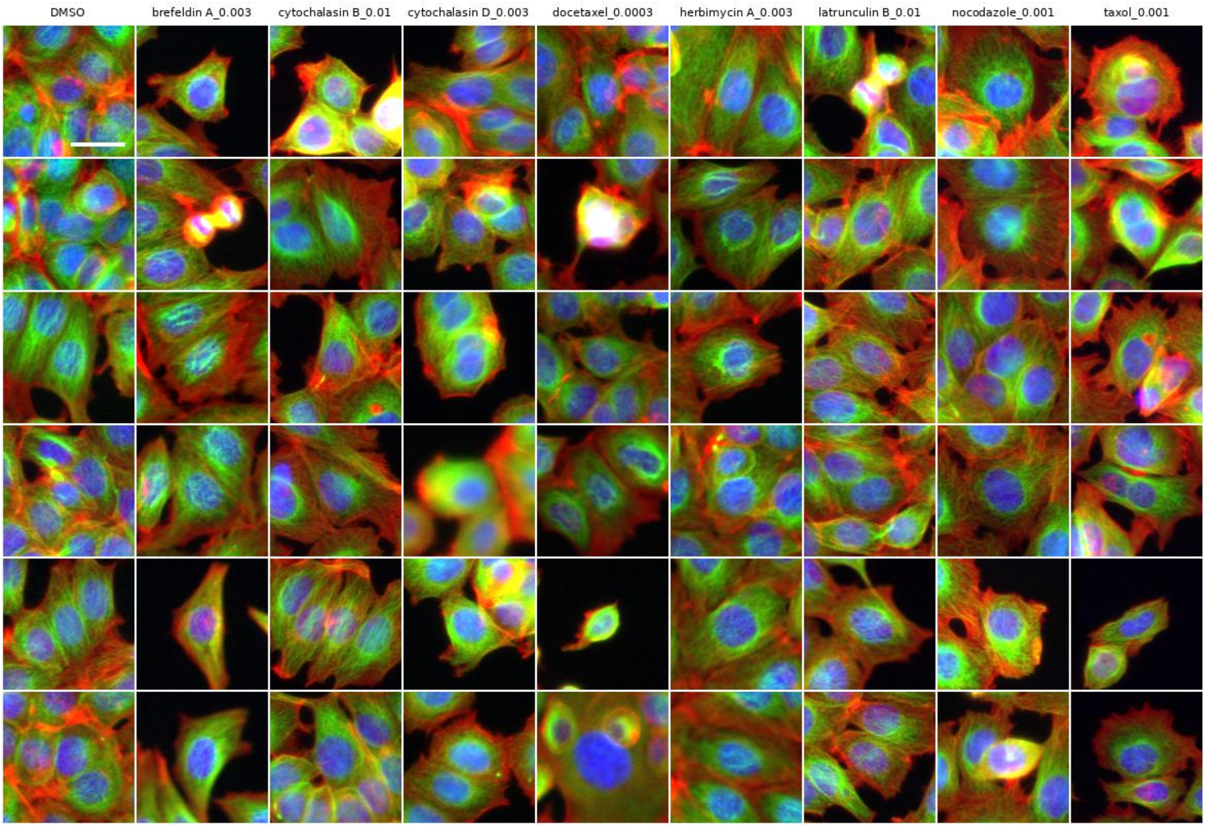


**Supplementary figure 15: Random samples of real cropped images of cells from low concentration compound treatments from the BBBC021 dataset.** Columns (=conditions) cannot be visually distinguished from one another (concentration unit is μM). Scale bar is 20μm.

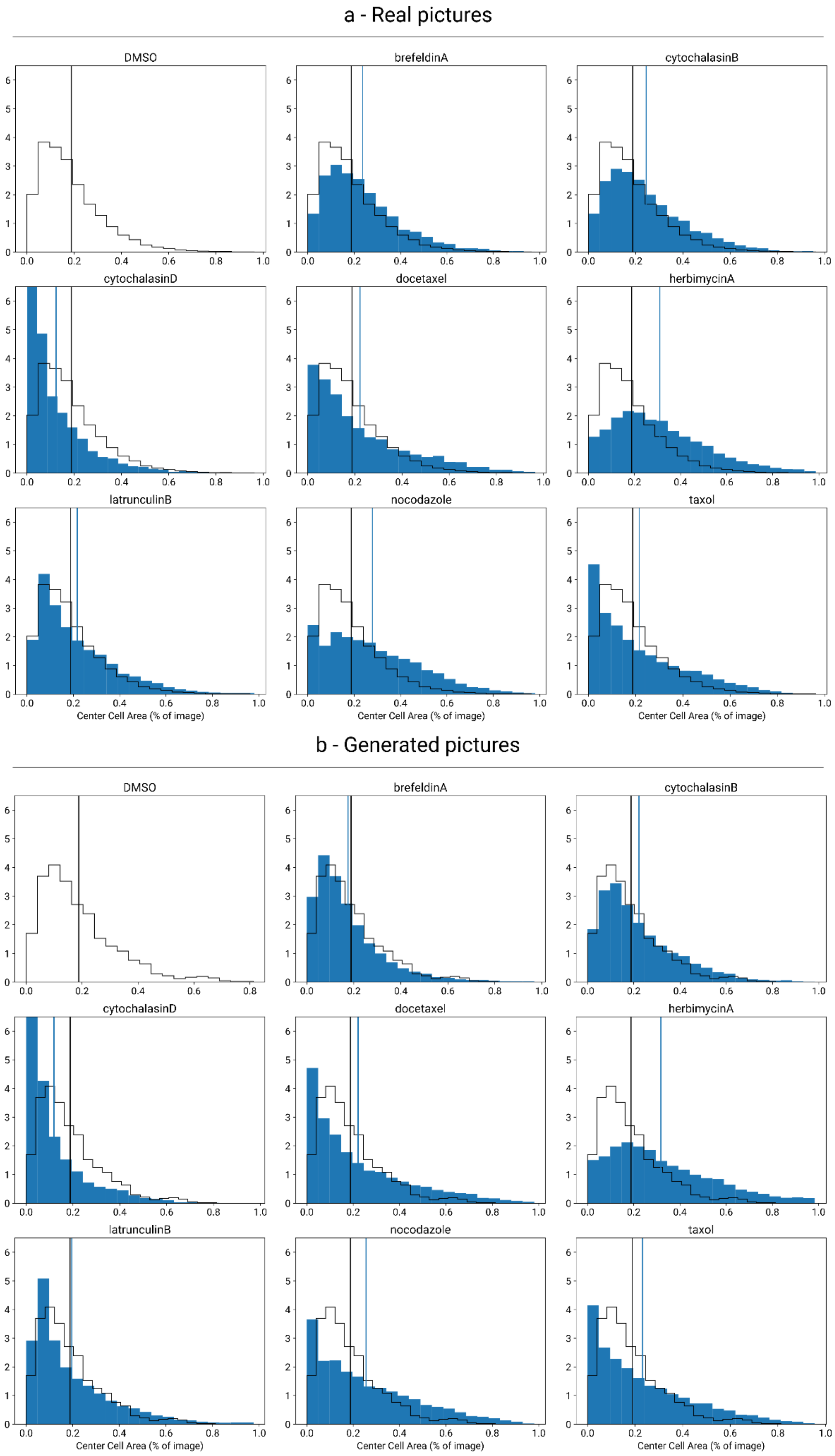
a - Real pictures
DMSO
brefeldinA
cytochalasinB
cytochalasinD
docetaxel
herbimycinA
latrunculinB
nocodazole
taxol
Center Cell Area (% of image)
b - Generated pictures
DMSO
brefeldinA
cytochalasinB
cytochalasinD
docetaxel
herbimycinA
latrunculinB
nocodazole
taxol
Center Cell Area (% of image)

**Supplementary figure 16: Distribution of the cell sizes for DMSO vs the lowest concentrations of various compounds (for a - real and b - generated images).** The distribution and the mean value (vertical line) for DMSO are in black. The distribution and the mean value for each compound are in blue.

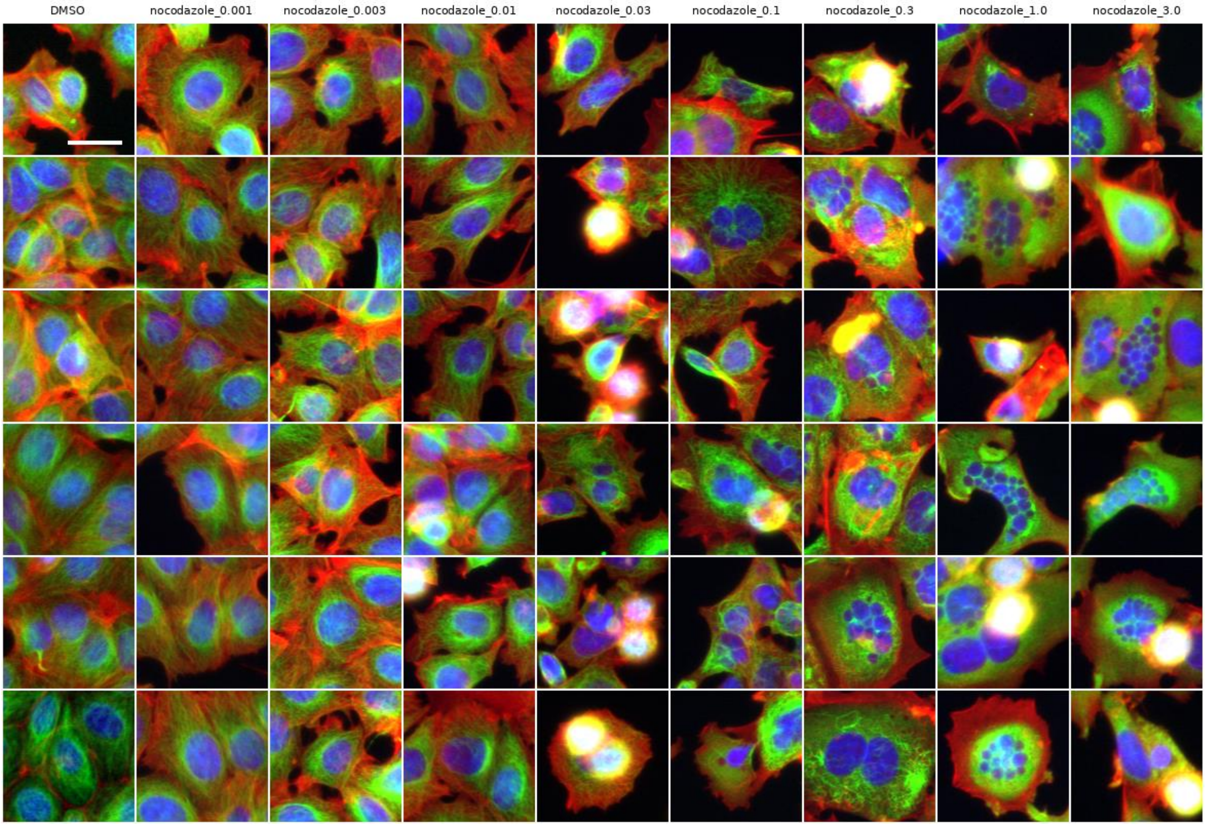


**Supplementary figure 17: Random samples of real cropped images of cells from each concentration of a dose response treatment of Nocodazole from the BBBC021 dataset**. While the effect of nocodazole is gradually visible, it is not necessarily homogeneous (concentration unit is µM). Scale bar is 20µm.

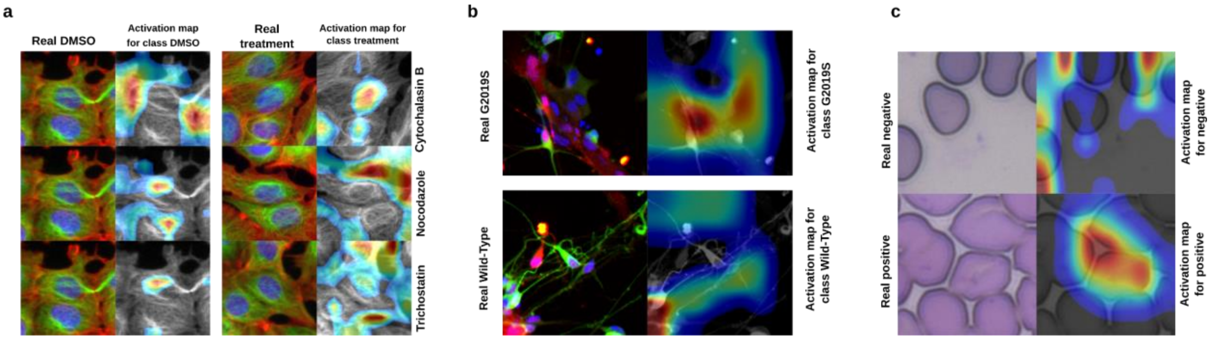


**Supplementary figure 18: Class Activation Maps (CAM).** CAM for classifiers trained on a - BBBC021 (image size is 128x128px, 24.3µm wide), b- LRKK2 (image size is 256x256px, 61µm wide) and c - Malaria (image size is 256x256px, 28.4µm wide) datasets. Activation Maps show which areas were considered important by the network, but they do not show precisely what specific difference of signal triggered discrimination, which, in case of subtle phenotype, makes interpretation impossible.

| Dataset | Concentration (μM) | Mean accuracy | SD accuracy |
|---|---|---|---|
| DMSO vs Brefeldin A | 0.003 | 0.83 | 0.02 |
| DMSO vs Brefeldin A | 10.0 | 0.97 | 0.03 |
| DMSO vs Cytochalasin B | 0.01 | 0.83 | 0.08 |
| DMSO vs Cytochalasin B | 30.0 | 0.94 | 0.01 |
| DMSO vs Cytochalasin D | 0.003 | 0.91 | 0.09 |
| DMSO vs Cytochalasin D | 10.0 | 0.97 | 0.02 |
| DMSO vs Docetaxel | 0.0003 | 0.83 | 0.03 |
| DMSO vs Docetaxel | 1.0 | 0.88 | 0.05 |
| DMSO vs Herbimycin A | 0.003 | 0.81 | 0.05 |
| DMSO vs Herbimycin A | 10.0 | 0.86 | 0.04 |
| DMSO vs Latrunculin B | 0.01 | 0.78 | 0.02 |
| DMSO vs Latrunculin B | 30.0 | 0.96 | 0.01 |
| DMSO vs Nocodazole | 0.001 | 0.82 | 0.04 |
| DMSO vs Nocodazole | 3.0 | 0.93 | 0.02 |
| DMSO vs Taxol | 0.001 | 0.86 | 0.00 |
| DMSO vs Taxol | 3.0 | 0.93 | 0.02 |
| Malaria (qPCR- vs qPCR+) | n/a | 0.74 | 0.03 |
| LRRK2 (G2019S vs WT) per tile | n/a | 0.63 | 0.03 |
| LRRK2 (G2019S vs WT) per sample | n/a | 1.00 | 0 |

**Supplementary Table 1: Accuracy of a basic CNN classifier on the datasets used in this paper.** For each dataset, we trained a classifier with 4 convolutional layers for 10 epochs using a 4-fold cross-validation, and computed the mean and standard deviation of the accuracy over these 4 runs. For the BBBC021 datasets, training was performed for the lowest and highest concentrations of each compound treatment. Even in the latter case, where differences could not be assessed by eye, a good accuracy could still be reached quickly, indicating that CNN can leverage invisible discriminative features. For the LRRK2 dataset, each well of a well plate was split in many tiles with the corresponding well annotation to feed the CNN. For the Malaria dataset, random tiles from each thin blood smear were used to feed the CNN with the corresponding thin blood smear annotation.

| Dataset | images | Conditions | FID (generated cells vs real cells) | FID (real cells vs real cells) | FID (generated cells vs real imagenet) | FID (real cells vs real imagenet) |
|---|---|---|---|---|---|---|
| BBBC021 | 117,191 | 73 | 1.08 | 0.279 +/- 0.003 | 218.7 | 221.2 |
| Translocation | 15,999 | 2 | 6.46 | 0.634 +/- 0.003 | 299.2 | 270.2 |
| Golgi | 134,180 | 2 | 4.34 | 0.136 +/- 0.003 | 281.3 | 268.4 |
| Malaria | 60,000 | 2 | 2.57 | 0.406 +/- 0.007 | 373.5 | 407.0 |
| LRRK2 | 70,520 | 2 | 3.47 | 0.334 +/- 0.002 | 252.6 | 220.0 |

**Supplementary Table 2: Number of images and conditions used for training, and Frechet Inception DIstances (FID)**. The first FID column is a regular FID computed between 50k generated images and the real dataset. The second FID column represents the FIDs computed between the first half and the second half of a real cell dataset (computed over 10 random splits to calculate the mean +/- standard deviation). This latter calculation provides the distribution of minimum FID values achievable for a given dataset. The third column are FIDs computed between the real cell images and a sample of 10k natural images taken from ImageNet. Finally, the last column are FIDs computed between generated cell images and a sample of 10k natural images taken from ImageNet. To further appreciate the FID values of cell images in this table please compare them to the scale we computed at various levels of image degradations (see **Supplementary Fig. 7**)

| **Assay** | **Condition** | **Dose** | **Distinct samples (wells)** | **Images** |
|---|---|---|---|---|
| LRRK2 | Wild-Type | | 30 | 52013 |
| | Mutation | | 30 | 52354 |
| Malaria | Positive | | 50 (slides) | 30000 |
| | Negative | | 50 (slides) | 30000 |
| Golgi | DMSO | | 288 | 89446 |
| | Nocodazole | | 288 | 85574 |
| Translocation | DMSO | | 40 | 8118 |

| | | | | |
|---|---|---|---|---|
| | TNF5 | | 40 | 7881 |
| BBBC021 | Brefeldin A | 0,003 | 3 | 1306 |
| | | 0,01 | 3 | 1429 |
| | | 0,03 | 3 | 1120 |
| | | 0,1 | 3 | 376 |
| | | 0,3 | 3 | 168 |
| | | 1 | 3 | 154 |
| | | 3 | 3 | 145 |
| | | 10 | 3 | 164 |
| | Cytochalasin B | 0,01 | 3 | 1481 |
| | | 0,03 | 3 | 1704 |
| | | 0,1 | 3 | 1645 |
| | | 0,3 | 3 | 1427 |
| | | 1 | 3 | 1353 |
| | | 3 | 3 | 483 |
| | | 10 | 3 | 786 |
| | | 30 | 3 | 470 |
| | Cytochalasin D | 0,003 | 3 | 905 |
| | | 0,01 | 3 | 844 |
| | | 0,03 | 3 | 580 |
| | | 0,1 | 3 | 529 |
| | | 0,3 | 3 | 790 |
| | | 1 | 3 | 644 |
| | | 3 | 3 | 615 |
| | | 10 | 3 | 544 |
| | DMSO | | 19 | 9765 |
| | Docetaxel | 0,0003 | 3 | 1462 |

| | | | | |
|---|---|---|---|---|
| | | 0,001 | 3 | 863 |
| | | 0,003 | 3 | 538 |
| | | 0,01 | 3 | 497 |
| | | 0,03 | 3 | 540 |
| | | 0,1 | 3 | 509 |
| | | 0,3 | 3 | 533 |
| | | 1 | 3 | 557 |
| | Herbimycin | 0,003 | 3 | 1426 |
| | | 0,01 | 3 | 1346 |
| | | 0,03 | 3 | 989 |
| | | 0,1 | 3 | 1224 |
| | | 0,3 | 3 | 1217 |
| | | 1 | 3 | 916 |
| | | 3 | 3 | 457 |
| | | 10 | 3 | 487 |
| | Latrunculin B | 0,01 | 3 | 1909 |
| | | 0,03 | 3 | 1927 |
| | | 0,1 | 3 | 2027 |
| | | 0,3 | 3 | 1420 |
| | | 1 | 3 | 988 |
| | | 3 | 3 | 1085 |
| | | 10 | 3 | 1028 |
| | | 30 | 3 | 845 |
| | Nocodazole | 0,001 | 3 | 1290 |
| | | 0,003 | 3 | 1456 |
| | | 0,01 | 3 | 1431 |
| | | 0,03 | 3 | 639 |

| | | | | |
|---|---|---|---|---|
| | | 0,1 | 3 | 565 |
| | | 0,3 | 3 | 445 |
| | | 1 | 3 | 446 |
| | | 3 | 3 | 451 |
| | Taxol | 0,001 | 3 | 1137 |
| | | 0,003 | 3 | 497 |
| | | 0,01 | 3 | 402 |
| | | 0,03 | 3 | 384 |
| | | 0,1 | 3 | 407 |
| | | 0,3 | 333 | 40680 |
| | | 1 | 3 | 404 |
| | | 3 | 3 | 448 |

**Supplementary Table 3: Number of replicats and images per condition for each dataset.** Samples are wells from well plates except for the Malaria dataset where each considered sample is a patient thin blood smear slide with 600 images acquired from each of them. Except for the Malaria and the LRRK2 datasets, image crops of 128x128 or 256x256 pixels are acquired around each nucleus from all the samples. For the LRRK2 assay images acquired on the border of the wells are discarded.
