# Supplementary material for: Revealing invisible cell phenotypes with conditional generative modeling
Source: Nat Commun. 2023 Oct 11;14:6386. doi: 10.1038/s41467-023-42124-6 (PMC10567685; doi:10.1038/s41467-023-42124-6)
Supplement: Supplementary file 2 — Reporting Summary [file 41467_2023_42124_MOESM2_ESM.pdf]

nature research

Corresponding author(s): Auguste Genovesio and Yong Jun Kwon

Last updated by author(s): Sep 19, 2023

Reporting Summary

Nature Research wishes to improve the reproducibility of the work that we publish. This form provides structure for consistency and transparency in reporting. For further information on Nature Research policies, see our Editorial Policies and the Editorial Policy Checklist.

Statistics

For all statistical analyses, confirm that the following items are present in the figure legend, table legend, main text, or Methods section.

n/a	Confirmed	
☐	☒	The exact sample size (*n*) for each experimental group/condition, given as a discrete number and unit of measurement
☐	☒	A statement on whether measurements were taken from distinct samples or whether the same sample was measured repeatedly
☐	☒	The statistical test(s) used AND whether they are one- or two-sided<br>*Only common tests should be described solely by name; describe more complex techniques in the Methods section.*
☒	☐	A description of all covariates tested
☒	☐	A description of any assumptions or corrections, such as tests of normality and adjustment for multiple comparisons
☐	☒	A full description of the statistical parameters including central tendency (e.g. means) or other basic estimates (e.g. regression coefficient) AND variation (e.g. standard deviation) or associated estimates of uncertainty (e.g. confidence intervals)
☐	☒	For null hypothesis testing, the test statistic (e.g. *F*, *t*, *r*) with confidence intervals, effect sizes, degrees of freedom and *P* value noted<br>*Give P values as exact values whenever suitable.*
☒	☐	For Bayesian analysis, information on the choice of priors and Markov chain Monte Carlo settings
☒	☐	For hierarchical and complex designs, identification of the appropriate level for tests and full reporting of outcomes
☐	☒	Estimates of effect sizes (e.g. Cohen's *d*, Pearson's *r*), indicating how they were calculated

Our web collection on statistics for biologists contains articles on many of the points above.

Software and code

Policy information about availability of computer code

Data collection	We designed a web interface to explore synthtetic data we generated: https://www.phenexplain.bio.ens.psl.eu/
Data analysis	The code of the method to reproduce our results can be found on github here: https://github.com/biocompibens/phenexplain with a permanent link of the version used in the paper on Zenodo (https://doi.org/10.5281/zenodo.8289732).

For manuscripts utilizing custom algorithms or software that are central to the research but not yet described in published literature, software must be made available to editors and reviewers. We strongly encourage code deposition in a community repository (e.g. GitHub). See the Nature Research guidelines for submitting code & software for further information.

Data

Policy information about availability of data

All manuscripts must include a data availability statement. This statement should provide the following information, where applicable:

- Accession codes, unique identifiers, or web links for publicly available datasets
- A list of figures that have associated raw data
- A description of any restrictions on data availability

We provide all datasets (40Gb) included in the paper in a Zenodo repository (https://doi.org/10.5281/zenodo.8287453). One of them is a subset of the BBBC021v1, an image set available from the Broad Bioimage Benchmark Collection (Caie et al. 2010; Ljosa, Sokolnicki, and Carpenter 2012). We generated all the other datasets.

Field-specific reporting

Please select the one below that is the best fit for your research. If you are not sure, read the appropriate sections before making your selection.

- [x] Life sciences
- [ ] Behavioural & social sciences
- [ ] Ecological, evolutionary & environmental sciences

For a reference copy of the document with all sections, see nature.com/documents/nr-reporting-summary-flat.pdf

Life sciences study design

All studies must disclose on these points even when the disclosure is negative.

Sample size	Sample size (image number) for each condition in each assay are provided in Supplementary Table 3 and described in he "Statistics & Reproducibility" section
Data exclusions	Data exclusion (mostly images at the border of wells for LRRK2) is discribed in the "Statistics & Reproducibility" section
Replication	Replication (distinct samples) for each condition in each assay are provided in Supplementary Table 3 and described in the "Statistics & Reproducibility" section
Randomization	Random samples mostly used for FID computation is discribed in the "Statistics & Reproducibility" section
Blinding	There was no blinding

Reporting for specific materials, systems and methods

We require information from authors about some types of materials, experimental systems and methods used in many studies. Here, indicate whether each material, system or method listed is relevant to your study. If you are not sure if a list item applies to your research, read the appropriate section before selecting a response.

Materials & experimental systems

n/a	Involved in the study	
	x	Antibodies
	x	Eukaryotic cell lines
x		Palaeontology and archaeology
x		Animals and other organisms
	x	Human research participants
x		Clinical data
x		Dual use research of concern

Methods

n/a	Involved in the study	
x		ChIP-seq
x		Flow cytometry
x		MRI-based neuroimaging

Antibodies

Antibodies used	LRRK2 assay:<br>-- primary: Rabbit anti-tyrosine hydroxylase(https://www.merckmillipore.com/KR/en/product/Anti-Tyrosine-Hydroxylase-Antibody,MM_NF-AB152) at 1:1500 dilution<br>- primary: anti-α-Synuclein (https://www.bdbiosciences.com/en-us/products/reagents/microscopy-imaging-reagents/immunofluorescence-reagents/purified-mouse-anti-synuclein.610787) at 1:1500 dilution<br>- secondary: anti rabbit - Alexa Fluor 488 (Goat anti Rabbit–Alexa fluor 488: A-11008 Life Technologies) at dilution 1:1000<br>- secondary: anti-mouse - Alexa Fluor 633 (Goat anti-mouse-Alexa Fluor 633: A-21050 Life Technologies) at dilution1:1000<br><br>NF-kB nuclear translocation assay:<br>- primary: Rabbit anti-NF-κB p65 (C-20) (sc-372, Santa Cruz Biotechnology) https://www.scbt.com/p/nfkappab-p65-antibody-c-20 at 1:200 dilution<br>- secondary: anti-rabbit - Alexa Fluor 488 (Donkey anti Rabbit-Alexa fluor 488 : A-21206, Invitrogen Life Technologies) at 1:1000 dilution
Validation	Antinbodies were commercially validated by the vendor

Eukaryotic cell lines

Policy information about cell lines

Cell line source(s)	The study on dopaminergic neurons only used existing, cultured iPSC lines deposited in the European Bank for Induced

	Pluripotent Stem Cells (EBiSC, https://cells.ebisc.org/) and listed in the Human Pluripotent Stem Cell Registry (hPSCreg, https://hpscreg.eu/). Post-mitotic neurons from those iPSCs were obtained by a commercial provider (Life & Brain GmbH, Bonn, Germany, doi: 10.1016/j.stemcr.2022.09.001). Detailed information for the used lines is available in hPSCreg (https://hpscreg.eu/cell-line/STBCi004-B).<br><br>HeLa cells obtained from the commercial provider American Type Tissue Collection (ATCC, CCL-2) stably expressing EGFP-CCR5 (CC chemokine receptor 5) as described in Boncompain et al. 2019 were kindly provided by F. Perez's team, UMR144, Institut Curie.<br><br>HCC1143 cancer cells were obtained from the commercial provider American Type Tissue Collection (ATCC, CRL-2321™) - https://www.atcc.org/products/crl-2321
Authentication	Human Pluripotent Stem Cell Registry (hPSCreg, https://hpscreg.eu/)
Mycoplasma contamination	We confirm there was no mycoplasma contamination.
Commonly misidentified lines<br>(See ICLAC register)	None of the lines we used are in this database.

Human research participants

Policy information about studies involving human research participants

Population characteristics	Participants of the Malaria survey are people of both sexes from one year and older living in Benin.
Recruitment	The malaria slides come from a survey carried out in the field in Benin with participants who gave their informed consent to participate.
Ethics oversight	The malaria study received approval from the institutional ethics committee of the Center for Research in Entomology of Cotonou n°023/CREC/CEI-CREC/SA

Note that full information on the approval of the study protocol must also be provided in the manuscript.
